# Comprehensive map of the regulatory network triggered by MET exon 14 skipping reveals important involvement of the RAS-ERK signaling pathway

**DOI:** 10.1038/s41419-025-08086-x

**Published:** 2025-11-03

**Authors:** Marie-José Truong, Geoffrey Pawlak, Jean-Pascal Meneboo, Shéhérazade Sebda, Marie Fernandes, Martin Figeac, Mohamed Elati, David Tulasne

**Affiliations:** 1 Univ. Lille, CNRS, Inserm, CHU Lille, Institut Pasteur de Lille, UMR9020 – UMR1277 - Canther - Cancer Heterogeneity, Plasticity and Resistance to Therapies, Target team, Lille, France; 2 Univ. Lille, CNRS, Inserm, CHU Lille, UMR9020 – UMR1277 - Canther - Cancer Heterogeneity, Plasticity and Resistance to Therapies, Disco team, Lille, France; 3 Univ. Lille, CNRS, Inserm, CHU Lille, Institut Pasteur de Lille, US 41 - UAR 2014 - PLBS, Lille, France

**Keywords:** Non-small-cell lung cancer, Growth factor signalling

## Abstract

The MET exon 14 skipping mutation (named METex14Del) described in lung cancer leads to prolonged activation of signaling pathways and aberrant cell responses, but the link between HGF signaling and cell responses remains unclear. A putative lung cancer regulatory network of influential transcription factors was constructed from the transcriptomes of lung cancer cell lines. Transcriptomic data from METex14Del-expressing cells, stimulated or not by HGF, were mapped onto this lung cancer reference network and revealed activation of a major regulatory node composed mainly by the highly influential transcription factors ETS1, FOSL1 and SMAD3. HGF activation of METex14Del receptor induced the expression and phosphorylation of these three master regulators and the expression of their predicted target genes involved in migration and invasion. All these molecular and biological effects were inhibited by trametinib, a MEK inhibitor, which was potentiated by combination with capmatinib, a MET inhibitor. New mapping with transcriptomic data from trametinib-treated METex14Del cells validated the key role of the RAS-ERK pathway signaling in the activation of ETS1, FOSL1 and SMAD3 regulators and the induction of their target genes in HGF-activated METex14Del receptor. Thus, we report an original and powerful strategy to uncover key regulators, including transcription factors that have not been widely described in METex14Del signaling, such as SMAD3. These factors are activated by specific signaling pathways and could provide a novel therapeutic strategy involving a combination of receptor and signaling inhibitors.

## Introduction

Receptor tyrosine kinases (RTKs) provide an interface between the extracellular and intracellular environments. Once activated by an extracellular growth factor, RTKs can activate intracellular signaling pathways that relay information within the cell, triggering various cell responses such as proliferation, differentiation, migration/invasion, and changes in the cell death/survival balance [1]. Signaling pathways transmit information to various cell compartments, including the nucleus, where regulation of gene expression is one of the most important mechanisms influencing cell responses [2]. The transcriptional program, which can be deciphered by transcriptomic analyses, provides important information about biological responses, as gene expression profiles can be linked to key biological processes. However, how signaling pathways are integrated at the transcriptional level remains elusive.

In some cancers, RTK activation by genomic alterations can lead to oncogene addiction, in which cancer cell growth and/or survival is dependent on a single oncogene [3]. In this situation, targeted therapies against RTKs, mainly tyrosine kinase inhibitors (TKIs), are effective and have profoundly improved patient care [4]. In addicted cancer cells, overactive RTKs induce aberrant signaling leading to a deregulated transcriptional program. Thus, understanding oncogene addiction requires a deep understanding of signaling pathway integration at the transcriptional level. In addition, the efficacy of targeted therapies against RTKs is limited by primary or acquired resistance, often supported by activation of alternative signaling pathways that can bypass the targeted inhibition [5]. In this situation, understanding signaling pathway integration at the transcriptional level is also an important issue.

MET, a member of the RTK family, is an emerging target in cancer. In lung cancer, the *MET* gene has been found to be mutated in approximately 3% of patients, with point mutations, deletions or insertions affecting the splice sites of exon 14. Such mutations result in in-frame skipping of this exon (METex14Del) [6, 7] and thus deletion of the juxtamembrane regulatory domain of the receptor [8, 9].

Experiments using ectopic expression of METex14Del or its reconstitution by genome editing in normal lung epithelial cells and cancer cell lines have shown that activated METex14Del induces enhanced and sustained activation of downstream signaling pathways, including the RAS-ERK and PI3K-AKT pathways [10–13]. In normal epithelial cells, METex14Del activation depends on stimulation by HGF, its high-affinity ligand, and the mutant receptor can promote experimental tumor growth only in humanized mice expressing human HGF [11]. Thus, in contrast to most RTK-activating mutations described to date, which result in ligand-independent RTK activation, MET exon 14 skipping does not eliminate MET receptor dependence on ligand stimulation. This suggests the existence of different activation states depending on ligand availability.

The juxtamembrane domain of MET contains several negative regulatory sites: (i) phosphorylated serine 985, involved in downregulation of MET tyrosine kinase activity [14,15], (ii) phosphorylated tyrosine 1003, involved in recruitment of the E3 ubiquitin ligase CBL and thus important for MET degradation [16–18], (iii) aspartic acid 1002, a caspase cleavage site involved in regulation of the death/survival balance [19–22]. Downregulation of MET signaling appears to be mediated primarily by the CBL binding site [10].

Activated METex14Del can also induce a much more complex transcriptional program than its WT counterpart. Gene ontology analyses are consistent with the migration and invasion responses induced by METex14Del [11]. However, this approach based on mRNA levels does not take into account the complexity of gene regulation, which involves the coordinated action of multiple transcription factors and co-factors (TF/co-TFs) and the influence of signaling pathways on their regulation.

The recently developed software solution CoRegNet [23], which can be visualized with the interactive tool Cytoscape Widget [24], is based on expression correlations between known cooperative TFs/co-TFs and target genes extracted from transcriptomic data and on described interactions between these regulators. CoRegNet has already proven its ability in complex organisms to identify 12 microglia-specific transcriptional regulators [25], 10 key regulators driving the transition in non-alcoholic liver disease [26], and five master regulators specific to rheumatoid arthritis synovial fibroblasts [27].

Here we constructed a lung cancer-specific regulatory network based on the aggregation of extensive transcriptomic data from lung cancer cell lines. This network was then used to dynamically model the network of METex14Del, stimulated or not by HGF. A “dialogue” between biological experiments and modeling confirmed the robustness of the proposed network and allowed an integrative view of METex14Del signaling from its downstream signaling pathways to gene expression, which in turn regulates cell responses.

## Results

### Transcriptome-based modeling of the putative regulatory network of HGF-stimulated METex14Del

The Cancer Cell Line Encyclopedia provided transcriptomic data for 206 lung cancer cell lines, including 135 NSCLC cell lines, 50 SCLC cell lines and 21 other cell lines. Of the 206 cell lines, 105 were derived from primary tumors and 101 from metastatic tumors. Using these gene expression data and the CoRegNet package [23], we constructed a lung cancer regulatory network and then enriched it with TF binding sites, ChIP-seq data and protein interactions found in the CoRegNet-embedded databases [28–31]. The lung cancer-specific regulatory network consists of 502 key regulators that potentially regulate 4143 target genes which are connected by 18,053 regulator-target gene regulatory interactions. The key regulators are connected by 2014 described protein-protein interactions (*p* < 1e-130) and 61,272 transcription factor binding sites on their target genes (*p* < 1e-300). CoRegNet can identify the most influential regulators based on their influence score (Supplementary Table 1) and co-regulatory pairs in our network that share common target genes (Supplementary Table 2).

**Fig. 1:**
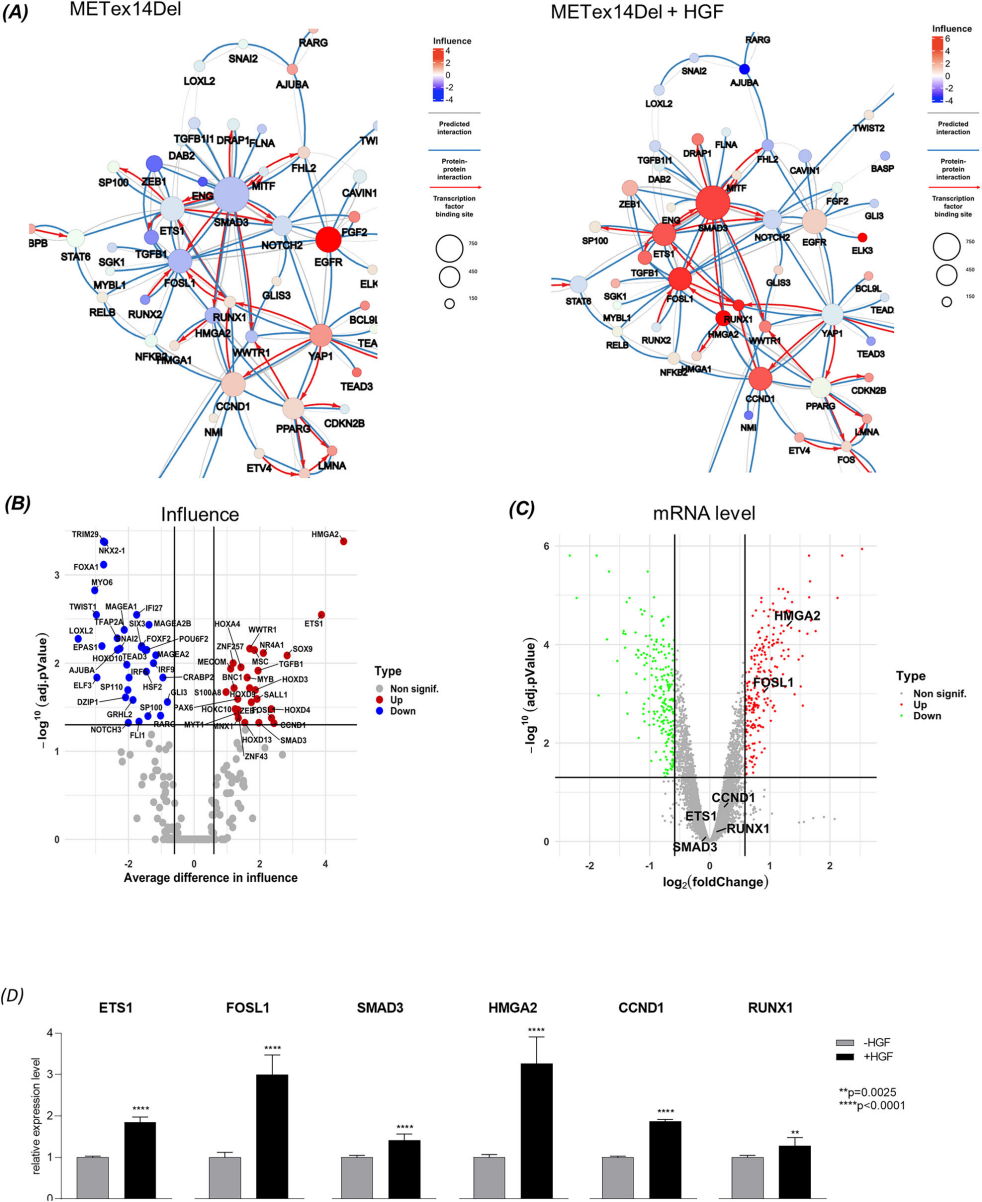
Regulatory network of 16HBE METex14Del in response to HGF. **A** Focus on the largest cluster of the co-regulatory network, showing the influence of TFs on METex14Del cells in the presence and absence of HGF. Circles represent DIRs and the radius of the circle is proportional to the number of target genes regulated by the DIR. Co-regulatory interactions between DIRs are indicated: protein-protein interactions with published evidence (blue lines), transcriptional regulation interactions with published evidence (red arrows), and interactions defined only by the h-LICORN algorithm (gray lines). **B** DIRS were visualized in a volcano plot from interference network analysis: average difference in influence vs. log10 transformed adj. *P*-value (regulators with an adj. *p*-value < 0.05 and an influence difference greater/smaller than 0.5 were considered up-/down-influenced (red/green). **C** DIRS were visualized in a volcano plot from transcriptomic analysis: log_2_ fold change vs. *p*-value of differential mRNA-level expression (genes with an absolute adj. *p*-value < 0.05 and a fold change greater/smaller than 1.5 were considered upregulated/downregulated (red/green)). **D** mRNA-level expression of the six main influent DIRs present in the putative regulatory node, in the presence and absence of HGF (triplicates of *n* = 3 independent experiments). Significance was determined by unpaired one-tailed *t*-test with Welch’s correction and data are expressed as mean ± SD.

To identify which of the TFs have a strong influence induced by HGF binding to the MET receptor, normalized expression levels of differentially expressed genes (DEGs) from transcriptomic datasets of 16HBE cells expressing MET WT (Supplementary Fig. S1A, B) or 16HBE METex14Del (Fig. 1A and Supplementary Fig. S1C, D) in the presence and absence of HGF [11], were mapped onto this lung cancer regulatory network. HGF-stimulated 16HBE cells expressing METex14Del reveal a large node of influential regulators formed by six positively interacting regulators: ETS1, FOSL1, SMAD3, HMGA2, CCND1 and RUNX1 with a high number of target genes (Fig. 1A and Supplementary Fig. S1D). ETS1, FOSL1 and SMAD3 interact with each other and binding sites were found in ETS1 and FOSL1 for SMAD3 and in SMAD3 for ETS1. The representation by the mean difference in influence (Fig. 1B) and the high value of the mean group influence (Supplementary Table 1) confirm the activation state of these six key regulators. Looking at the mRNA levels for these six regulators in the transcriptomic data, only FOSL1 and HMGA2 appeared to be significantly up-regulated under HGF stimulation (Fig. 1C), whereas by quantitative RT-PCR all six regulators showed significantly increased mRNA expression in the presence of HGF, with a stronger up-regulation for FOSL1 and HMGA2 (Fig. 1D).

### Evidence for a regulatory relationship between ETS1, FOSL1 and SMAD3 and their predicted target genes

**Fig. 2:**
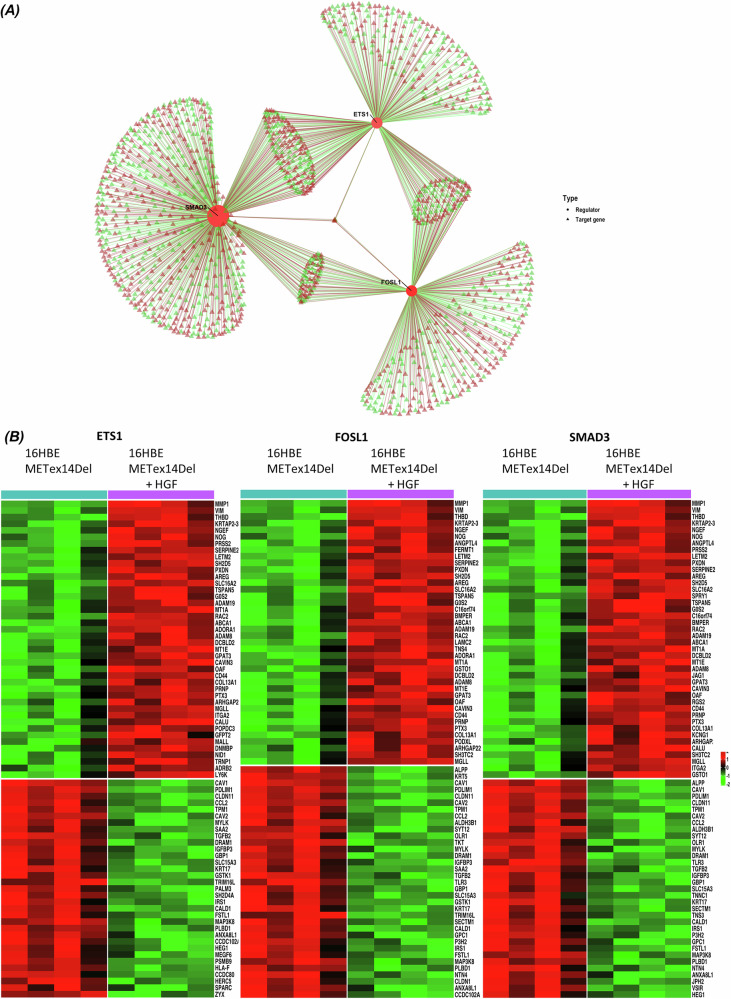
Identification of differentially expressed target genes potentially regulated by ETS1, FOSL1, and SMAD3 on 16HBE METex14Del cell line. **A** Representations of the transcriptional regulatory network of ETS1, FOSL1 and SMAD3 with their putative influence on positively (red triangle) and negatively (green triangle) regulated DEGs. **B** Heatmaps showing putative ETS1, FOSL1 and SMAD3 target genes based on METex14Del vs. HGF-activated METex14Del (adj *p*-value < 0.05 and absolute fold change >1.5) (*n* = 4 for each conditio*n*). Colors indicate high (red) and low (green) relative expression levels.

**Fig. 3:**
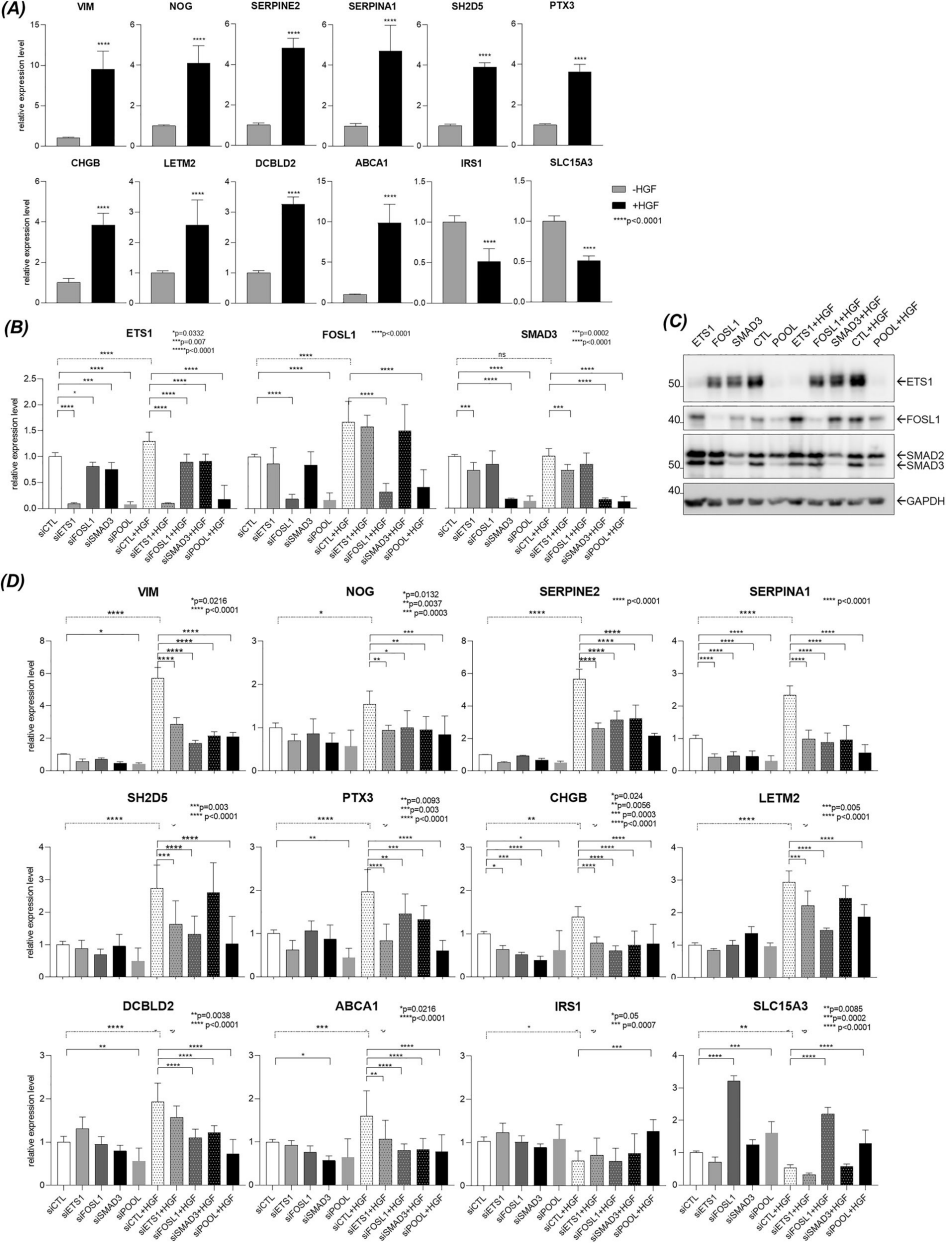
Effects of ETS1, FOSL1 and SMAD3 silencing on the expression of their predicted target genes on 16HBE METex14Del cell line. **A** The mRNA-level expression of selected target genes up- or down-regulated in response to HGF was determined by RT-qPCR (triplicates of *n* = 3 independent experiments). Significance was determined by unpaired one-tailed *t*-test with Welch’s correction and data are expressed as mean ± S.D. **B, C** The silencing efficacy of each specific siRNA (siETS1, siFOSL1, siSMAD3), used individually or in combination (siPOOL) was assessed by **B** RT-qPCR for mRNA-level expression (triplicates of *n* = 3 independent experiments) and **C** by Western blotting for protein-level expression (representative results). **D** mRNA-level expression of up- and down-regulated target genes induced by knockdown of ETS1, FOSL1 and SMAD3 expression (triplicates of *n* = 2, 3 or 4 independent experiments). Significance was determined by one-way ANOVA test and data are expressed as mean ± SD in panels **B** and **D**.

We then focused on the three most influential TFs: ETS1, FOSL1 and SMAD3. The transcriptional regulatory network of 16HBE METex14Del constructed with the microarray datasets allowed visualizing the putative influence of ETS1, FOSL1 and SMAD3 on their respective or common target genes (Fig. 2A). The 50 most regulated targets are shown in a heatmap (Fig. 2B), and the complete list of regulated target genes by specific or combined TFs was compiled in Supplementary Table 2. The predicted differential expression by HGF activation was confirmed at the mRNA expression level for ten HGF up-regulated (VIM, NOG, SERPINE2, SERPINA1, SH2D5, PTX3, CHGB, LETM2, DCBLD2, ABCA1) and two HGF down-regulated (IRS1, SLC15A3) target genes (Fig. 3A) in 16HBE METex14Del cell line. Similar results were obtained for ten up-regulated (ADMA19, KRTAP2-3, G0S2, NGEF, COL13A1, THBD, TSPAN5, ARHGAP22, CAVIN3, RAC2) and two down-regulated (CAV1, CLDN11) additional target genes (Supplementary Fig. S2).

To assess the regulatory relationships between ETS1, FOSL1 and SMAD3 and their targets, these TFs were silenced by RNA interference, separately or in combination, and the effect on expression of 12 original target genes was evaluated. The efficacy of each siRNA pair and the pooled one was confirmed at the mRNA level (Fig. 3B) and at the protein level (Fig. 3C), under stimulation or not by HGF in 16HBE METex14Del cell line. Among the HGF upregulated target genes, VIM, NOG, SERPINE2, SERPINA1, PTX3, CHGB, ABCA1 and LETM2 showed reduced expression in the presence of the three silencers, alone or in combination (Fig. 3D). The effects of ETS1 silencing on PTX3 expression and FOSL1 silencing on LETM2 expression were particularly pronounced. Reduced expression of SH2D5 was observed with either ETS1 or FOSL1 silencing alone and with the three silencers in combination. Reduced expression of DCBLD2 was observed with either the FOSL1 or SMAD3 silencer used alone and with the three silencers combined. Of the two downregulated genes, IRS1 and SLC15A3 showed restored expression in the presence of the three silencers. SLC15A3 expression was strongly increased in the presence of the FOSL1 silencer alone (Fig. 3D).

### Silencing of ETS1, FOSL1 and SMAD3 slightly affects wound healing and scattering

**Fig. 4:**
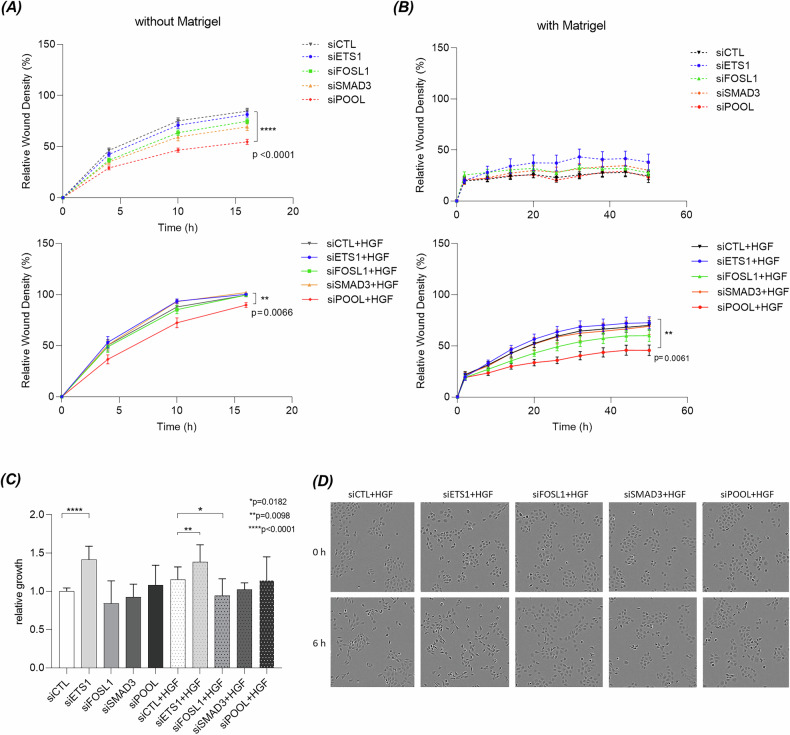
Effect of ETS1, FOSL1, and SMAD3 silencing on HGF-induced migration, invasion, proliferation and scattering on 16HBE METex14Del cell line. Forty-eight h after transfection with siRNAs targeting ETS1, FOSL1 and SMAD3 alone or in combination, wound healing was monitored without (**A**) or with (**B**) Matrigel in the presence and absence of HGF (triplicates of *n* = 4 independent experiments). In all graphs, only statistically significant differences between the negative control (siCTL) and the applied treatment (an siRNA used alone or pooled with the other siRNAs, siPOOL) in the presence or absence of HGF are indicated. Wound healing data are expressed as mean ± SEM and significance was determined by two-way ANOVA test. **C** The effect of siRNA on proliferation was determined by Alamar blue staining after 16 h of stimulation with HGF and relative growth was expressed as relative mean fluorescence (6 replicates of *n* = 4 independent experiments). Proliferation data are expressed as mean ± SD and significance was determined by one-way ANOVA test. **D** Images of the effect of silencing on cell scattering were captured at different time points using the Incucyte system and representative images from baseline and 6 h after stimulation with HGF are shown.

Knockdown of ETS1, FOSL1 or SMAD3 alone in 16HBE METex14Del cell line did not affect wound healing, but combined knockdown slightly reduced both basal and HGF-stimulated responses (Fig. 4A). Similarly, in the presence of Matrigel, combined knockdown of all three TFs reduced HGF-induced wound invasion (Fig. 4B). HGF did not increase cell proliferation, but ETS1 knockdown enhanced this process both in the presence and absence of HGF (Fig. 4C). In addition, ETS1 knockdown appeared to increase HGF-induced scattering, whereas FOSL1 and SMAD3 knockdown decreased it (Fig. 4D). These results show that combined knockdown of the three TFs slightly reduces HGF-induced migration, invasion and scattering of 16HBE METex14Del cell line.

### The RAS-ERK signaling pathway controls ETS1, FOSL1 and SMAD3 phosphorylation and cellular wound healing

**Fig. 5:**
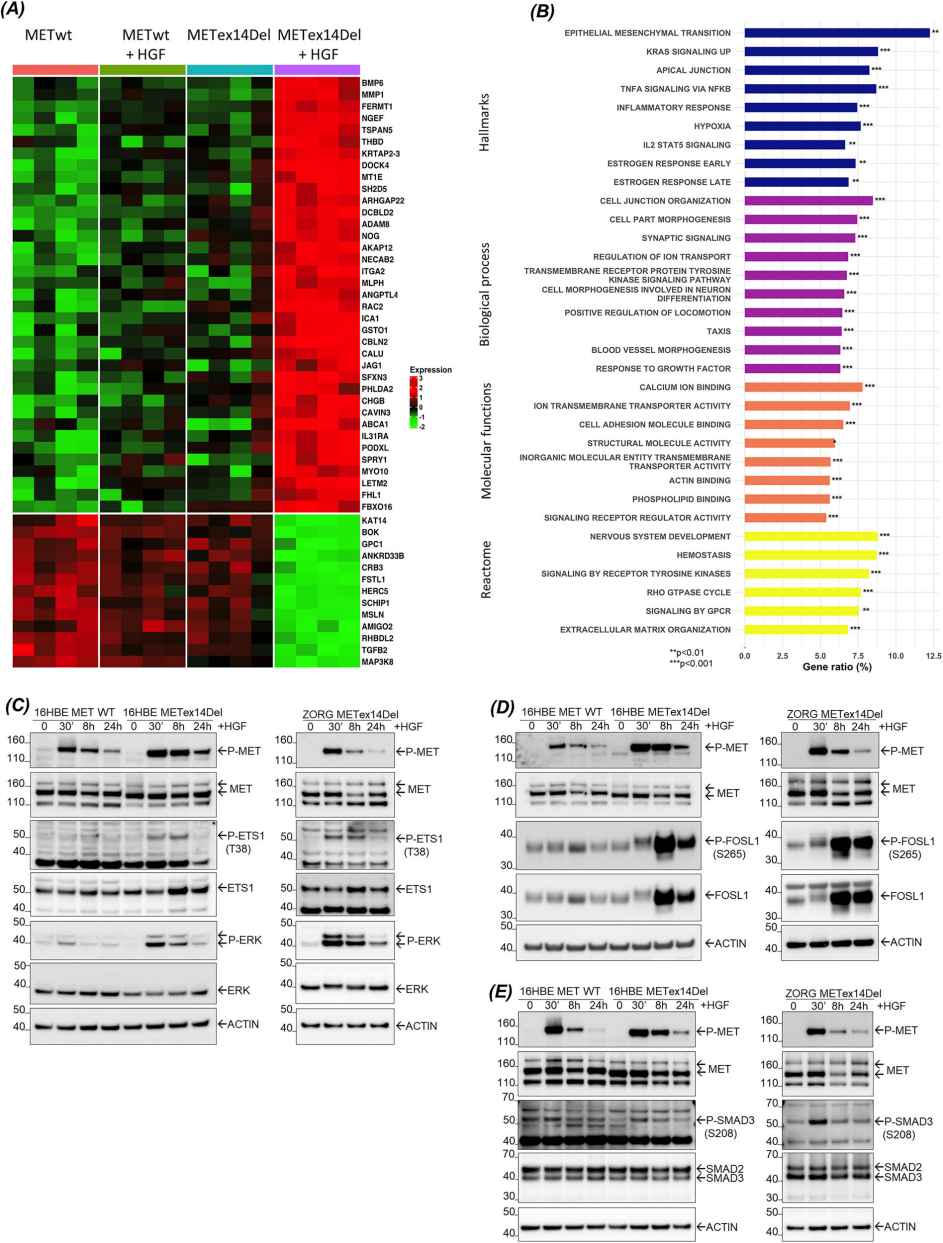
Gene ontology (GO) annotation of target genes induced by HGF activation of METex14Del. **A** Heatmap of the 50 most significantly differentially regulated target genes in response to HGF (adj. *p*-value < 0.05 and absolute fold change >1.5) between indicated conditions (*n* = 4 per condition). Colors indicate high (red) and low (green) levels of relative expression. **B** Shown are gene ratios (%) of GO enrichment for the differentially expressed target genes according to Hallmarks, Biological Process, Molecular Functions and Reactome annotations. Overrepresentation analysis was performed using the hypergeometric test: **p-value < 0.01, ***p-value < 0.001. **C–E** The 16HBE cells expressing MET WT or METex14Del and ZORG cells derived from lung cancer patients expressing METex14Del were stimulated with HGF in a time course experiment. Expression and activation of **C** ETS1 and its form phosphorylated at T^38^ (P-ETS1), **D** FOSL1 and its form phosphorylated at S^265^ (P-FOSL1), and **E** SMAD3 and its form phosphorylated at S^208^ (P-SMAD3) were analyzed by Western blotting (representative results).

Since ETS1, FOSL1 and SMAD3 silencing did not fully inhibit known biological functions induced by HGF, we investigated upstream regulators to understand how these 3 TFs might act together in the regulatory node. The heatmap of the top 50 differentially expressed target genes of 16HBE MET WT vs. 16HBE METex14Del upon HGF stimulation (Fig. 5A) and the complete gene ontology (GO) enrichment analysis revealed a significant overrepresentation of annotations such as epithelial-mesenchymal transition (EMT), KRAS signaling up or positive regulation of locomotion (Fig. 5B and Supplementary Table 3). Given our previous study showing HGF/MET signaling induces activation of ETS1 through ERK-dependent phosphorylation of its threonine 38 (Thr^38^) [32, 33], we examined the induction of ETS1, FOSL1 and SMAD3 phosphorylation upon stimulation by HGF (Fig. 5C–E). As expected, ETS1 was found to be phosphorylated at Thr^38^ in 16HBE METex14Del cells, an effect detected up to 8 h after stimulation. No phosphorylation of this TF was detected in their WT counterparts (Fig. 5C). Similar sustained ETS1 phosphorylation was observed in ZORG cells (Fig. 5C) and H596 cells (Supplementary Fig. S3A) derived from NSCLC patients harboring the METex14Del variant. A slight increase in ETS1 expression was observed in METex14Del cells 8 h after HGF stimulation (Fig. 5C). A strong induction of FOSL1 expression and phosphorylation at Ser^265^ was found in our three cell models peaking at 8 h post-simulation and persisting until 24 h (Fig. 5D and Supplementary Fig. S3B). SMAD3 was phosphorylated at Ser^208^ in our three cell models with a maximum phosphorylation at 30 min post-stimulation (Fig. 5E and Supplementary Fig. S3C). The expression of ETS1, FOSL1, SMAD3 mRNA was also assessed in a similar HGF time course stimulation. In 16HBE METex14Del, ZORG and H596 cells, HGF stimulation induced weak ETS1 mRNA expression, which was only detected at 8 h post-stimulation. FOSL1 mRNA expression increased from 30 min post-stimulation, reaching a maximum expression at 8 h and a decrease at 24 h in all cell lines. Expression was stronger in 16HBE METex14Del cells compared to its WT counterpart. SMAD3 mRNA expression remained unchanged during the course of HGF stimulation in any of the cell lines (Supplementary Fig. S3D–G). Taken together, the mRNA expression of ETS1, FOSL1 and SMAD3 is consistent with their protein expression.

**Fig. 6:**
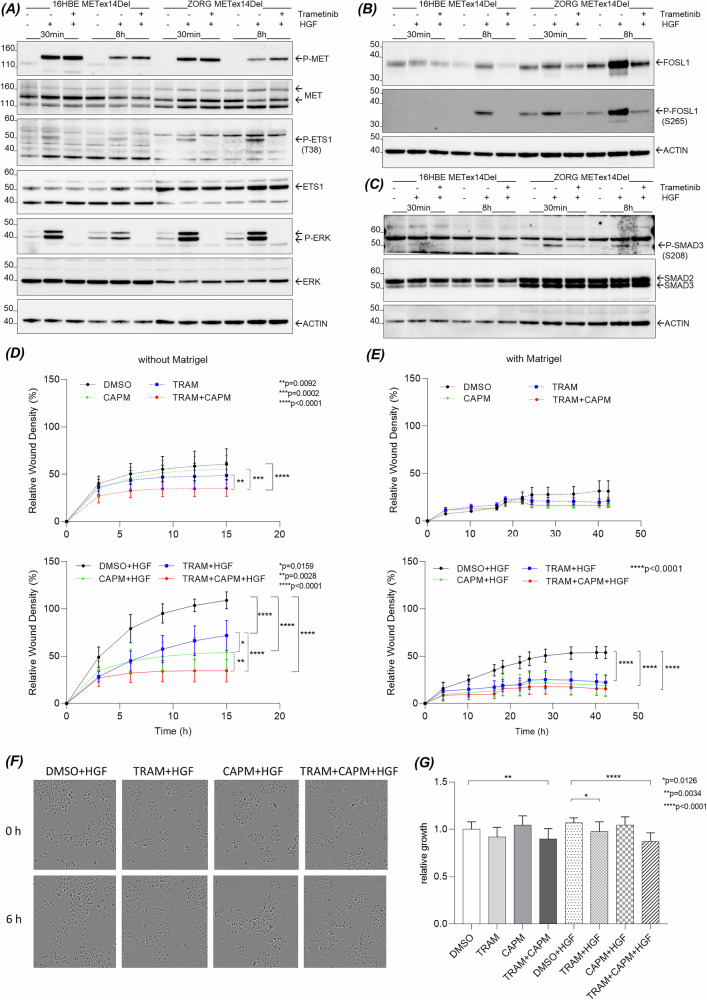
Effect of MEK inhibitor on ETS1, FOSL1 and SMAD3 phosphorylation and cell responses induced by HGF. The effect of trametinib (TRAM, a MEK inhibitor) on the expression of (**A**) P-ETS1/ETS1, **B** P-FOSL1/FOSL1 and **C** P-SMAD3/SMAD3 in 16HBE METex14Del cells and ZORG cells stimulated or not with HGF was determined by Western blotting (representative results). The effects of trametinib and capmatinib (CAPM, a MET inhibitor), alone and in combination, on 16HBE METex14Del cell wound healing were evaluated without **D** and with **E** Matrigel (6 replicates of *n* = 3 independent experiments); **F** scattering in 16HBE METex14Del cells stimulated or not with HGF (representative photo of baseline and 6 h after inhibition under HGF) and **G** proliferation (6 replicates of *n* = 4 independent experiments). In all graphs, only statistically significant differences between the negative control without HGF (DMSO) and the different treatments (individual or combined inhibitors) in the presence or absence of HGF are indicated. Wound healing data in panels (**D, E**) are expressed as mean ± SEM and significance was determined by two-way ANOVA test. In panel **G**, proliferation data are expressed as mean ± SD and significance was determined by one-way ANOVA test.

To confirm involvement of the RAS-ERK signaling, trametinib was used to specifically inhibit MEK. The addition of trametinib completely reversed the HGF-induced phosphorylation of ETS1 at Thr^38^, FOSL1 at Ser^265^ and SMAD3 at Ser^208^ in 16HBE METex14Del and ZORG cells (Fig. 6A–C). U0126, another MEK inhibitor, was also effective in the 2 cell models (Supplementary Fig. S4A–C). The effect of trametinib on the biological responses of 16HBE METex14Del cells was also investigated. Trametinib was found to inhibit HGF-induced wound healing both in the absence (Fig. 6D) and presence of Matrigel (Fig. 6E), although less effectively than the MET TKI capmatinib used as a control. Co-treatment with both capmatinib and trametinib resulted in more potent inhibition (Fig. 6D–F). Similar results were observed in cell scattering assays (Fig. 6F). Cell proliferation was only slightly affected by the co-treatment (Fig. 6G). Wound healing experiments in presence or absence of Matrigel have been also performed in both ZORG and H596 lung cancer cells and confirmed the inhibitory effect of the capmatinib and trametinib (Supplementary Fig. S5A–D).

To determine whether another oncogene driver activates the regulatory node composed of ETS1, FOSL1 and SMAD3, four cell lines harboring an activating mutation in the EGFR gene were treated with Osimertinib (a third generation EGFR TKI) or trametinib. As expected, the four cell lines displayed constitutive EGFR and ERK phosphorylation, which was inhibited by osimertinib. Trametinib inhibited ERK phosphorylation but not EGFR (Supplementary Fig. S6A, B). ETS1 was found to be constitutively phosphorylated in PC9, HCC0827 and H1975 cells and this was inhibited by both osimertinib and trametinib. ETS1 expression was not detected in H3255 cells (Supplementary Fig. S6A). FOSL1 expression and phosphorylation were inhibited by both inhibitors in PC9 and HCC0827 cells. In H1975 cells, the inhibitors did not reduce FOSL1 expression or phosphorylation and in H3255 cells, FOSL1 expression and phosphorylation were weak and remained unchanged by treatment (Supplementary Fig. S6B). SMAD3 was found to be phosphorylated in H1975 and H3255 cells and was inhibited by both osimertinib and trametinib (Supplementary Fig. S6B). Overall, only one or two transcription factors were found to be phosphorylated and inhibited by both inhibitors in each different cell line.

### Involvement of the RAS-ERK signaling pathway in the positive regulation of the METex14Del regulatory network

**Fig. 7:**
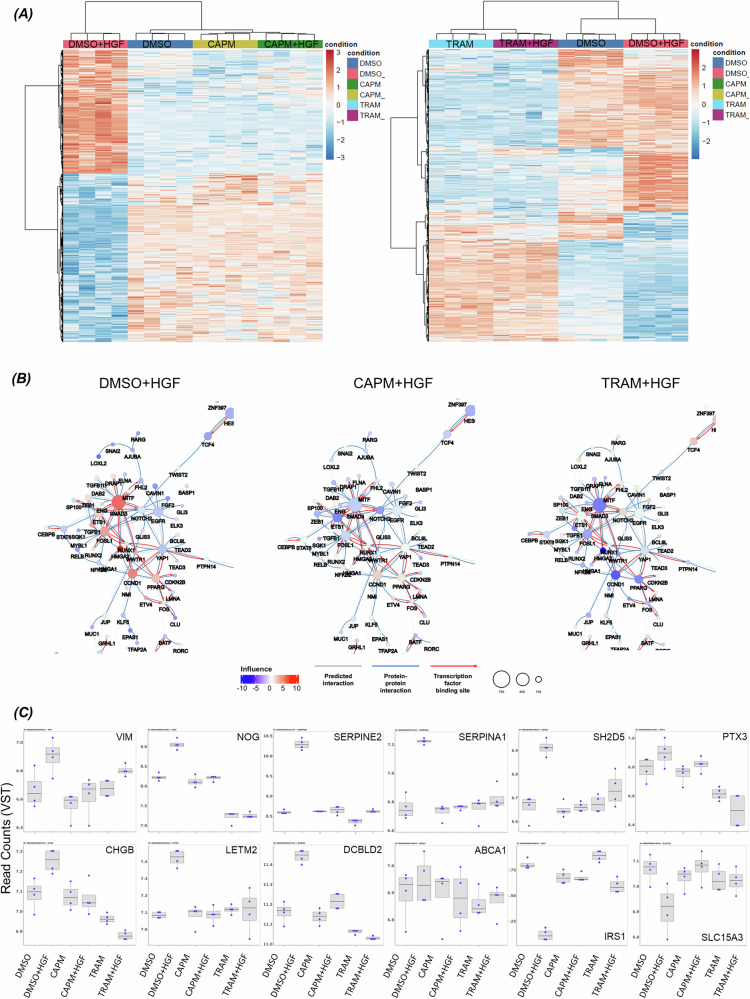
Effect of MET and MEK inhibitors on the transcriptional program induced by HGF and on the predicted regulatory network. **A** Heatmaps of differentially expressed genes (adj. *p*-value < 0.05 and absolute fold change > 1.5) in 16HBE METex14Del cells treated with capmatinib (CAPM) or trametinib (TRAM) and stimulated or not with HGF are shown (*n* = 4 per condition). **B** Zooms on the major regulatory node of the co-regulatory network of METex14Del cells in the presence and absence of inhibitors (CAPM, TRAM) upon stimulation with HGF. The radius of the circle is proportional to the number of target genes of the respective DIR. Co-regulatory interactions between transcription factors are indicated: protein-protein interactions with published evidence (blue line), transcriptional regulation interactions with published evidence (red arrow), and interactions defined only by the h-LICORN algorithm (gray line). **C** Expression of selected target genes regulated by ETS1, FOSL1, SMAD3 are shown in box-and-whisker plots. The box plot shows the 25th, 50^th^, and 75th percentiles, while the blue dots show the variant stabilization transformation (VST) of gene expression for each sample (*n* = 4 per condition). The median is indicated by the line across the box.

A new RNA-seq transcriptomic analysis was performed on 16HBE METex14Del cells stimulated or not with HGF and treated or not with capmatinib (CAPM) or trametinib (TRAM). The heatmap of CAPM treatment shows a restoration of the transcriptional program corresponding to the unstimulated state (Fig. 7A) and the heatmap of TRAM treatment shows a drastic change in all differentially expressed genes under both basal and HGF-stimulated conditions (Fig. 7A). To go further, these RNAseq datasets were mapped onto the lung cancer-specific reference network (Fig. 7B and Supplementary Fig. S7) and showed that HGF treatment alone resulted in an activated network profile similar to Fig. 1A, with activation of the same six positively influential TFs of the major regulatory node: ETS1, FOSL1, SMAD3, HMGA2, CCND1 and RUNX1 (Fig. 7B). As expected, CAPM completely inhibited their positive influence and stronger effect was found with TRAM treatment (Supplementary Table 4). Furthermore, when the expression of the previously analyzed regulated target genes of ETS1, FOSL1 and SMAD3 were plotted in a box-and-whisker plot (Fig. 7C), VIM, NOG, SERPINE2, SERPINA1, SH2D5, CHGB, LETM2, DCBLD2, IRS1 and SLC15A3 showed the expected up- or down-regulation under HGF stimulation and were reversed by these two inhibitor treatments. The exception was ABCA1, which showed only weak activation (Fig. 7C).

## Discussion

Disease phenotypes, including those related to disease progression and response to therapy, are maintained by small groups of TFs and co-TFs [34, 35]. Therefore, it is important to focus on the identification of these regulators. There are several bioinformatic methods for inferring gene regulatory networks from high-throughput data to enable the discovery of disease-related genes and/or pathways. To date, successful approaches for reverse-engineered construction of context-specific networks have been ARACNe, LICORN or GENIE3 with enrichment of interaction evidence such as protein-protein interactions and/or transcriptional regulation [36]. Our regulatory network concept is based on the representation of numerous TFs and co-regulators with an indication of cooperation/interaction [23, 37] and also the level influence of regulators on target genes [23].

Our reverse engineering approach to infer the first lung cancer-specific gene regulatory network from the lung cancer cell dataset without any a priori knowledge allowed us to distinguish two different states of cell activation: HGF-stimulated or not. Notably, we and others have demonstrated that METex14Del activation requires HGF stimulation in various cell lines expressing METex14Del, via genome editing [8, 11, 38], by ectopic expression [10, 39] or cells derived from patients [11, 40]. The dependence of tumor formation in vivo on HGF has been demonstrated using human 16HBE METex14Del cells, which induce tumor growth only when xenografted in HGF-humanized mice. Interestingly, mice infected with a lentivirus expressing mouse METex15del (equivalent to human METex14Del) also develop lung tumors. However, the contribution of HGF to tumorigenesis was not elucidated in this experiment, as mouse HGF could also contribute to tumor growth [8]. By contrast, cells displaying both MET exon 14 skipping and MET gene amplification, such as Hs746T, receptor activation is ligand-independent, likely due to the high expression of MET. Nevertheless, the ligand dependence of METex14Del remains debated, regardless of its overexpression, since receptor phosphorylation and downstream signaling activation were observed without ligand stimulation in some models in which METex14Del was reconstituted by genome editing [13].

Our transcriptomic data of HGF-stimulated METex14Del, interpretated by the regulatory network, revealed three interconnected transcription factors: FOSL1, ETS1 and SMAD3, the last two only highlighted by the network representation but not by transcriptomic analysis. Furthermore, the network showed that the relationship between ETS1, SMAD3 and FOSL1 was characterized by protein-protein interactions between them and by the fact that the transcription factors ETS1 and SMAD3 have binding sites on each other’s genes and FOSL1 has a binding site for SMAD3. Several studies have already documented interactions between these differentially influential regulators (DIRs): co-regulation of gene expression by ETS1 and SMAD3 [41–43] and the existence of active transcriptional complexes containing them [44]; an association between FOSL1 and SMAD3 leading to co-regulation of target genes has also been demonstrated [45], as has a potential cooperation between ETS1, SMAD3 and AP1 to regulate the angiopoietin-like 4 gene [46]. Therefore, although the interplay between these transcription factors had already been documented, our study shows that they actively cooperate in the context of an integrated biological response (HGF stimulation of the METex14Del receptor) to regulate a set of target gene. This demonstrates that they belong to the regulatory node.

Transcription factors can be activated by several mechanisms, including expression, phosphorylation state, localization, and interaction with co-activators. In our model, we found that mRNA and protein expression of FOSL1 is strongly increased under HGF stimulation, but ETS1 and SMAD3 expression is only weakly increased. This suggests a different activation mechanism for the latter two, independent of protein expression, as confirmed here by the phosphorylation/activation state for the 3 TFs under HGF stimulation. Note that ETS1 and FOSL1 phosphorylations are known to be ERK-dependent [33, 47–49] and can be induced by HGF stimulation in cells expressing the WT form of MET [32, 50]. SMAD3 phosphorylation can also be ERK-dependent, on serine residues within the linker domain [51] and plays different roles depending on the cell context [52]. As expected, direct blockade of the MET receptor with capmatinib did not allow HGF/MET to induce these stimulations and blocking only the RAS-ERK signaling pathway with trametinib prevented the activation of some specific HGF-activated DIRs and the expression of related target genes involved in cell motility and invasion. Lung cancer regulatory networks from trametinib-treated cells confirmed the strong involvement of the RAS-ERK signaling pathway and its positive influence on ETS1, FOSL1 and SMAD3. However, METex14Del is able to induce sustained activation of other signaling pathways including PI3K/AKT, which should also be investigated. The network also highlights the potential involvement of a variety of other TFs, such as YAP1 of the HIPPO pathway, which is known to be involved in tumorigenesis [53]. Epithelial-to-mesenchymal transition (EMT) is an important step in cell migration and invasion, specifically through reduction of intercellular adhesion, loss of apical-basal polarity and gain of motility [54] notably upon binding of TGFβ, HGF and EGF to their respective receptors. ETS1, FOSL1 and especially SMAD3 are known to induce EMT [55–57]. In our study, several EMT target genes tested (VIM, SERPINE2, PTX3 and ITGA2) were found to be regulated by HGF and their expression was reduced by knockdown of the combination of the 3 TFs. Functionally, significant inhibition of METex14Del-induced cell motility and invasion was achieved by knocked down of ETS1, FOSL1 and SMAD3, suggesting that these three regulators work together to regulate migration and invasion. However, additional transcription factors may be involved including HMGA2, ZEB1 and TWIST2, known to be involved in lung cancer [54, 58].

Taken together, our transcriptomics-based regulatory network and the functional experiments performed to support our interpretations highlight a number of key transcription factors that act together to regulate the biological responses induced by METex14Del. In particular, inhibition of a single TF of the node did not perturb the biological responses, and only simultaneous knockdown of three of them resulted in significant but not complete, inhibition. Furthermore, inhibition or restoration of target gene expression required knockdown of two or three of them. Thus, the regulation of genes by multiple TFs forming an interconnected node could ensure the robustness of the network, with the knock-out of one regulator being insufficient to disrupt the biological outcome. To go further, we interpreted “influence” as post-transcriptional activation, i.e. phosphorylation, of the transcription factors, rather than simply increased expression. This led us to identify the RAS-ERK pathway as an important signaling pathway that can positively regulate both the entire major regulatory node and associated biological responses. Interestingly, activation of ETS1, FOSL1 or SMAD3 was also observed in several cell lines harboring mutation of EGFR. However, activation of these three transcription factors was not found together in the same cell type. This suggests that the complexity and specificity of the regulatory network activated by an oncogene driver depends on the expression or availability of key regulatory factors. Therefore, importantly, unlike the TFs, this pathway is readily targeted by pharmacological approaches, opening the way to novel therapeutic strategies, particularly in the context of incomplete response to MET TKIs in patients harboring METex14Del mutations.

## Materials and methods

### Cell lines

The parental 16HBE cell line (MET WT) and derived cells (METex14Del) [11] were maintained in GIBCO MEM. The ZORG cell line was derived from a pleural effusion of a MET-TKI resistant patient and were maintained in RPMI1640 [59]. The H596, PC9 (EGFR del E746_A750), HCC0827 (EGFR del E746_A750), H1975 (EGFR T790M L858R) and H3255 (EGFR L858R) cell lines were obtained from the American Type Culture Collection (Manassas, VA) and were maintained in RPMI1640. All media were supplemented with 10% FBS (Sigma-Aldrich, Merck KGaA, Darmstadt). All cells were routinely tested for mycoplasma using MycoAlertTM (Lonza, Basel, Switzerland). Cells were cultured at 37 °C under a humidified controlled atmosphere of 5% CO2 in air. For MET activation experiments, cells were starved in serum-free medium for 16 h after overnight adherence. Cells were then pretreated with inhibitors for 3 h before activation by HGF for 30 min, 8 h or 24 h.

### RNA interference

The ETS1 (VHS40612 and VHS40614), FOSL1 (HSS188462 and s15585), and SMAD3 (VHS41114 and VHS41111) siRNAs and the Stealth siRNA negative control Lo GC (12935200) and Silencer® Select Negative Control #1 siRNA (#4390843) were purchased from Thermo Fischer Scientific. Transfections were performed using Lipofectamine™ RNAiMAX (Thermo Fisher Scientific) with 50 nM siRNA per well according to manufacturer’s protocol. Cells were used for further experiments 48 h after transfection.

### Immunoblotting

The Nucleospin RNA/Protein Kit (Macherey-Nagel, Düren, DE) was used to extract RNA and proteins from siRNA validation experiments. All other proteins were extracted with RIPA buffer supplemented with protease and phosphatase inhibitor cocktail (1% aprotinin, 1 mM PMSF, 1 µM leupeptine, 1 mM Na3VO4, 20 mM β-glycerophosphate), sonicated for 20 s, and centrifuged at 15000 rcf for 15 min. Protein samples was quantified using BCA protein assay kit (Thermo Fischer Scientific) and equal amounts of protein were resolved on NuPAGE Bis-Tris polyacrylamide gel (Invitrogen, Thermo Fischer Scientific) and transferred to Immobilon-P polyvinylidene fluoride membranes (Merk Millipore, Darmstadt, DE). After blocking with casein buffer (0.2% casein in PBS containing 0.1% Tween 20), the membranes were sequentially incubated with the indicated primary antibodies and HRP-conjugated secondary antibodies for 1 h before detection (Supplementary Table 5). The chemiluminescence signal was detected using SuperSignal West Dura or Femto (ThermoFisher Scientific) and captured using LAS 3000 (Fujifilm, Ratingen, DE). For reprobing, primary antibodies were removed from membranes using Antibody stripping buffer (Gene Bio-Application, Israel) according to manufacturer’s instructions. Representative results of *n* = 3 independent experiments are shown. The full length uncropped original western blots used in the manuscript are provide as part of their original submission (Supplemental Material file).

### RT-qPCR analysis

After total RNA extraction using the Nucleospin RNA/Protein Kit (Macherey-Nagel) and quantification using NanoDrop™ 2000 (Thermo Scientific), cDNA was reverse transcribed using the High Capacity cDNA Reverse Transcriptase Kit (Invitrogen). Quantitative PCR was performed using Fast SYBR Green Master Mix on QuantStudio3 (Applied Biosystems, Thermo Fisher Scientific). All conditions were performed in triplicate of *n* = 2, 3 or 4 independent experiments (see figure comments). Cycle threshold (ΔΔCt) values were calculated by normalization to β2 m and the gene expression levels were compared using the 2-ΔΔCt method. Primers sequenced are listed in Supplementary Table 6.

### Scratch wound healing assay

After 24 h of attachment, cells (transfected or not) were either starved with 0.1% FBS medium for 4 h or treated with mitomycin C (Sigma-Aldrich) for 2 h (ZORG) before scratch wounds were made using a 96-well WoundMaker (Essen BioScience, Sartorius, Göttingen, Germany) according to manufacturer’s instructions. For the invasion assay, 50 µl of Matrigel matrix was added to each well and allowed to solidify for 30 min in a 37 °C CO2 incubator. Cells were then treated with or without inhibitor in the presence or absence of HGF in 0.1% FBS medium or in 10% FBS medium (ZORG) (*n* = 3 to 4 independent experiments; see figure comments). Images of the wounds were captured automatically in the CO2 incubator using IncuCyte Zoom or SX5 software (Essen BioScience) every 2 h. Data were analyzed for wound confluence and calculated using the IncuCyte software package.

### Proliferation and scattering cell imaging

After 24 h of attachment, cells (transfected or not) were starved for 4 h in 0.1% FBS medium before treatment or not with inhibitor in the presence or absence of HGF for 16 h. Cell growth and viability were quantified using AlamarBlue Cell Viability Reagent (Invitrogen) and fluorescence was measured using a Multiskan RC Microplate Reader (ThermoLabsystems) with 560/590 (ex/em) wavelength filter settings. Each result was expressed as the average optical density (6 replicates of *n* = 3 to 4 independent experiments; see figure comments). For scattering, images were acquired automatically in the CO2 incubator using the IncuCyte system every 2 h.

### Inference of gene regulatory networks

The Cancer Cell Line Encyclopedia project [60] provides transcriptomic data for lung cancer cell lines. The Human Transcription Factor Catalog [61] and the TcoF-DB v2 database [62] provided a list of 2 375 regulators corresponding to 1 639 TFs with experimentally validated DNA binding specificity and 752 co-TFs. Human transcription factor binding sites were modeled using MotifDB R/Bioconductor [63] and promoter sequences were searched using the PWMEnrich R/Bioconductor package [64]. ChIP-seq data from the ChEA2 database [65] and the tftargets R package and various databases (e.g. TRED, ITFP, ENCODE, BEDOPS, TRRUST) provided all regulatory evidence from TF to target gene, resulting in a list of 2 256 674 TF-target interactions. The list of TF-TF cooperation containing 1 257 053 protein-protein interactions from different databases (e.g. BioGrid, HIPPIE, STRING, and HPRD) was obtained using the R package iRefR [66]. Gene regulatory networks were inferred using the bioconductor package CoRegNet [23]. Starting with transcriptomic data and a list of human regulators, CoRegNet uses the hLICORN algorithm [37] to capture regulatory interactions between regulators and target genes.

### Quantification of regulatory influence signals

The regulatory network structure provides a set of genes that are activated or repressed under reference conditions. Based on this structure, we can capture the influence of each regulator, a latent signal of regulator activity in each sample based on its observed effect on downstream entities. For each regulator, Welch’s t-test was performed to compare the distribution of activated *(A*^*r*^*)* and repressed *(I*^*r*^*)* genes. The influence of a regulator r is calculated as Influence(r) = (¯(E(A^r))-¯(E(I^r)))/√((μ_(A^r)^2)/A^r +(μ_(I^r)^2)/I^r) where *E(A*^*r*^*)* and *E(*$I$^*r*^*)* are the expressions of the activated and repressed genes in the samples, respectively, ¯(E(A^r))∧¯(E(I^r)) are their respective means, and *μA*^*r*^ and *μI*^*r*^ are their standard deviations. A regulator is active only if it activates *A*^*r*^ and represses *I*^*r*^ according to the expectations of the regulatory network model, resulting in a positive *t*-value in the Welch *t*-test. The *influence* of a regulator is only present if it activates or represses at least five genes. In the case that expression values of target genes might be missing in some of the tumor samples, their expression values were estimated using the LatNet method [67], with expression levels in cell lines as a reference dataset.

### Identification of group-specific regulators and target genes

Gene expression analysis was performed on the normalized gene expression dataset and DIRs were identified using the linear models for microarray data (LIMMA) R package [68], and *p*-values were adjusted using the Benjamini-Hochberg method. Regulators with an adj. *p*-value < 0.05 were considered DIRs.

### Functional enrichment analysis of DEGs

GO enrichment analysis was performed on Hallmarks, Molecular Function and Reactome using the R package msigdbr [69], with overrepresentation analysis methods using the R packages clusterProfiler [70] and enrichR [71].

### Library construction and sequencing

3’RNA-Seq libraries were prepared using the QuantSeq 3’ mRNA-Seq Library Prep Kit FWD (Lexogen, Greenland, US) with 200 ng of total RNA according to manufacturer’s instructions. Libraries are purified and loaded onto a High Sensitivity DNA Chip controlled by an Agilent Bioanalyzer 2100 (Agilent, Santa Clara, CA). The concentration and size distribution of the libraries are checked. Each library is pooled equimolarly, and the final pool is sequenced on a Nova 6000 instrument (Illumina, San Diego, CA) with 100 cycles of chemistry.

### Data analysis for transcriptome data

The *fastp* program was used to remove poor quality regions and poly(A) from the reads, and only reads with quality score threshold of 20 and reads above 25 pb were retained. Read alignments were performed using the *STAR* program with the human genome reference (GRCh38) and reference gene annotations (*Ensembl*). The UMI (Unique Molecular Index) allowed us to reduce errors and biases in the PCRs, which were processed using *fastp* and *umi tools*. Based on the read alignments and the UMI, we counted the number of molecules per gene using *FeatureCount* (from 3.73 M to 11.9 M molecules, average 6.82 M). Other programs used for read quality control and workflow were Q*ualimap*, *fastp*, *FastQC* and *MultiQC*. Differential gene expression of RNA-seq was performed using the *R/Bioconductor* package *DESeq2*. The cut-off for differentially expressed genes was adj. *p*-value < 0.05 and absolute fold change >1.5.

### Statistics

All results except Network inference and transcriptomic analysis are expressed as mean ± SEM. or SD for the indicated number of independent experiments. Data were analyzed using GraphPad Prism® 9 (San Diego, CA).

## Supplementary information

Supplementary Fig. S1

Supplementary Fig. S2

Supplementary Fig. S3

Supplementary Fig. S4

Supplementary Fig. S5

Supplementary Fig. S6

Supplementary Fig. S7

Supplementary Table 1

Supplementary Table 2

Supplementary Table 3

Supplementary Table 4

Supplementary Table 5

Supplementary Table 6

Legends of supplementary figures and tables

revised original western blots

## Data Availability

Transcriptomic data from 16HBE cells expressing MET WT or METex14Del, stimulated or not by HGF, are available in the Sequence Read Archive under accession number PRJNA764905 (GSE184514, raw data). The RNAseq data from 16HBE with or without treatment (CAPM/TRAM) were deposit in the Sequence Read Archive (SRA) under accession number PRJNA1123466.
