# Supplementary figures and images for: Comprehensive map of the regulatory network triggered by MET exon 14 skipping reveals important involvement of the RAS-ERK signaling pathway
Source: Cell Death Dis. 2025 Nov 3;16(1):783. doi: 10.1038/s41419-025-08086-x (PMC12583649; doi:10.1038/s41419-025-08086-x)

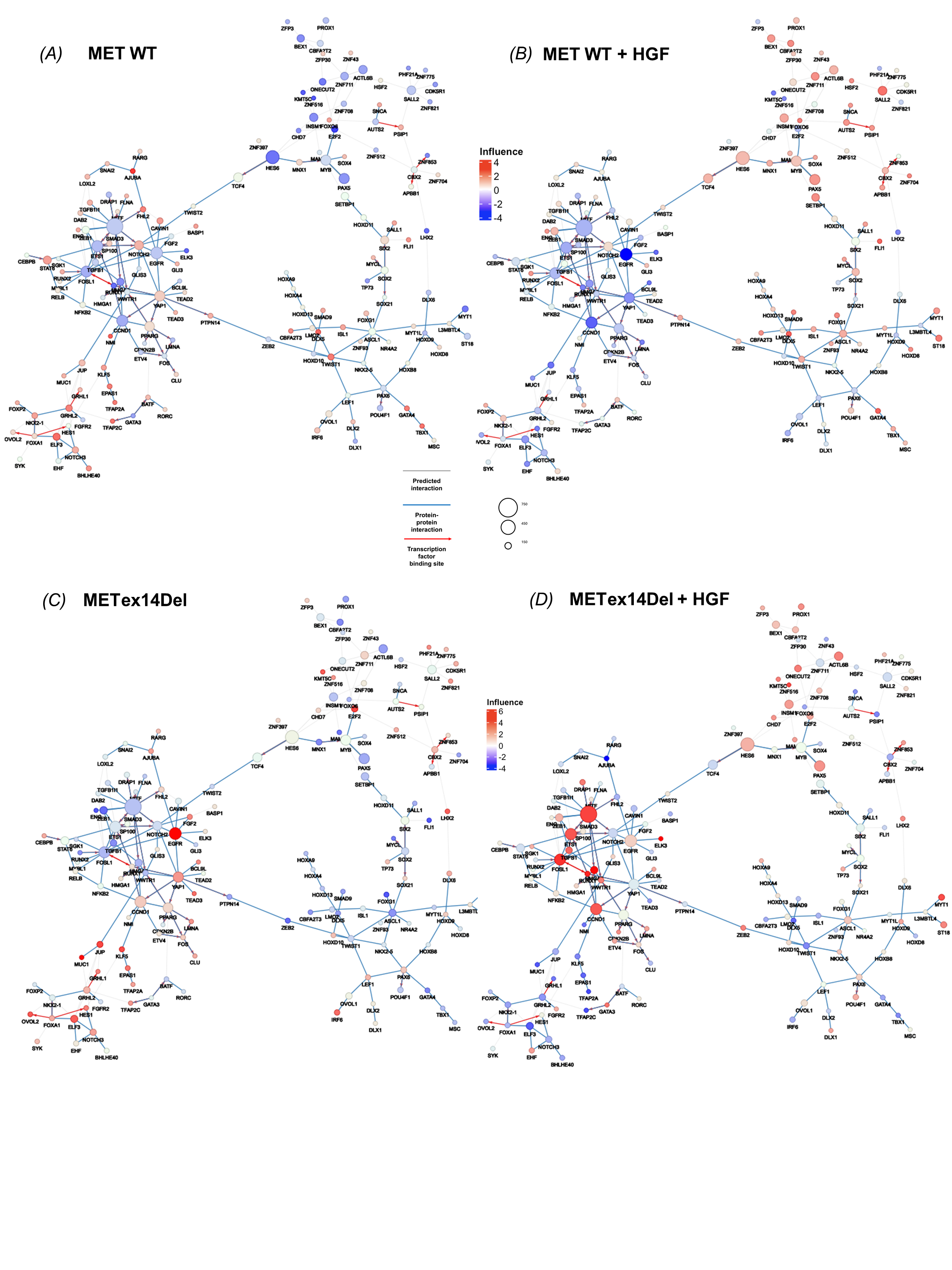

Supplement: Supplementary file 1 — Supplementary Fig. S1 [file 41419_2025_8086_MOESM1_ESM.tif]

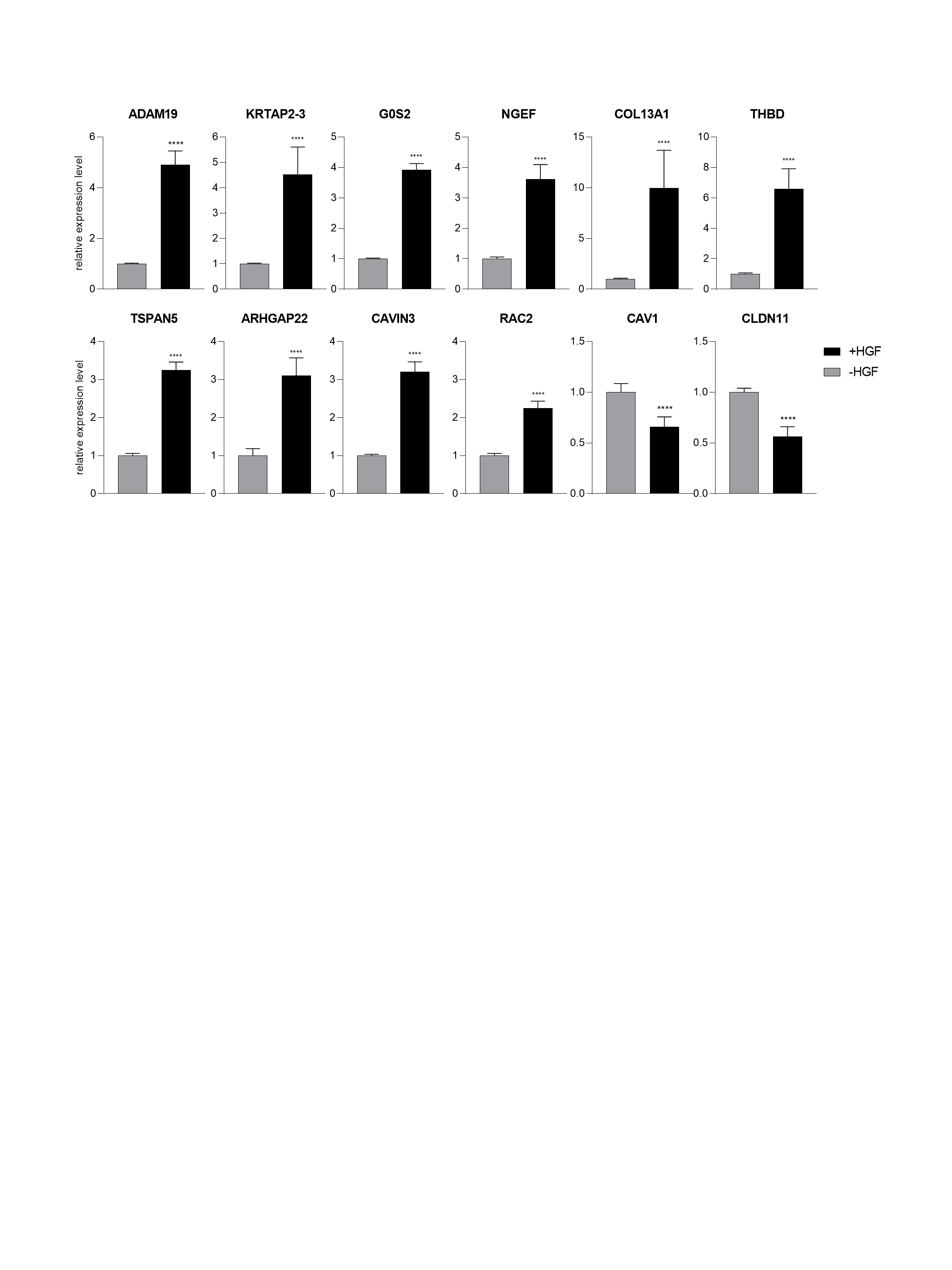

Supplement: Supplementary file 2 — Supplementary Fig. S2 [file 41419_2025_8086_MOESM2_ESM.tif]

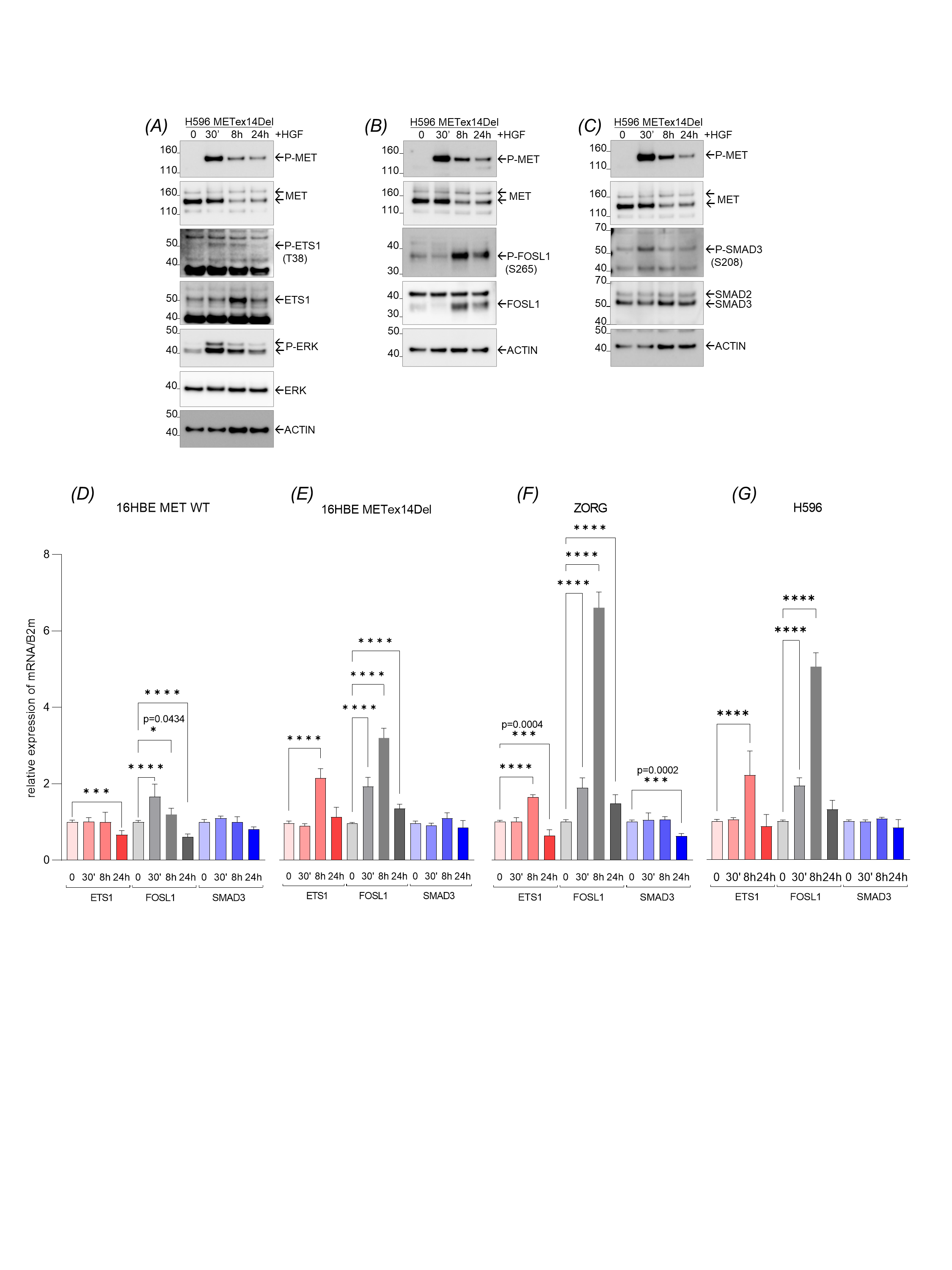

Supplement: Supplementary file 3 — Supplementary Fig. S3 [file 41419_2025_8086_MOESM3_ESM.tif]

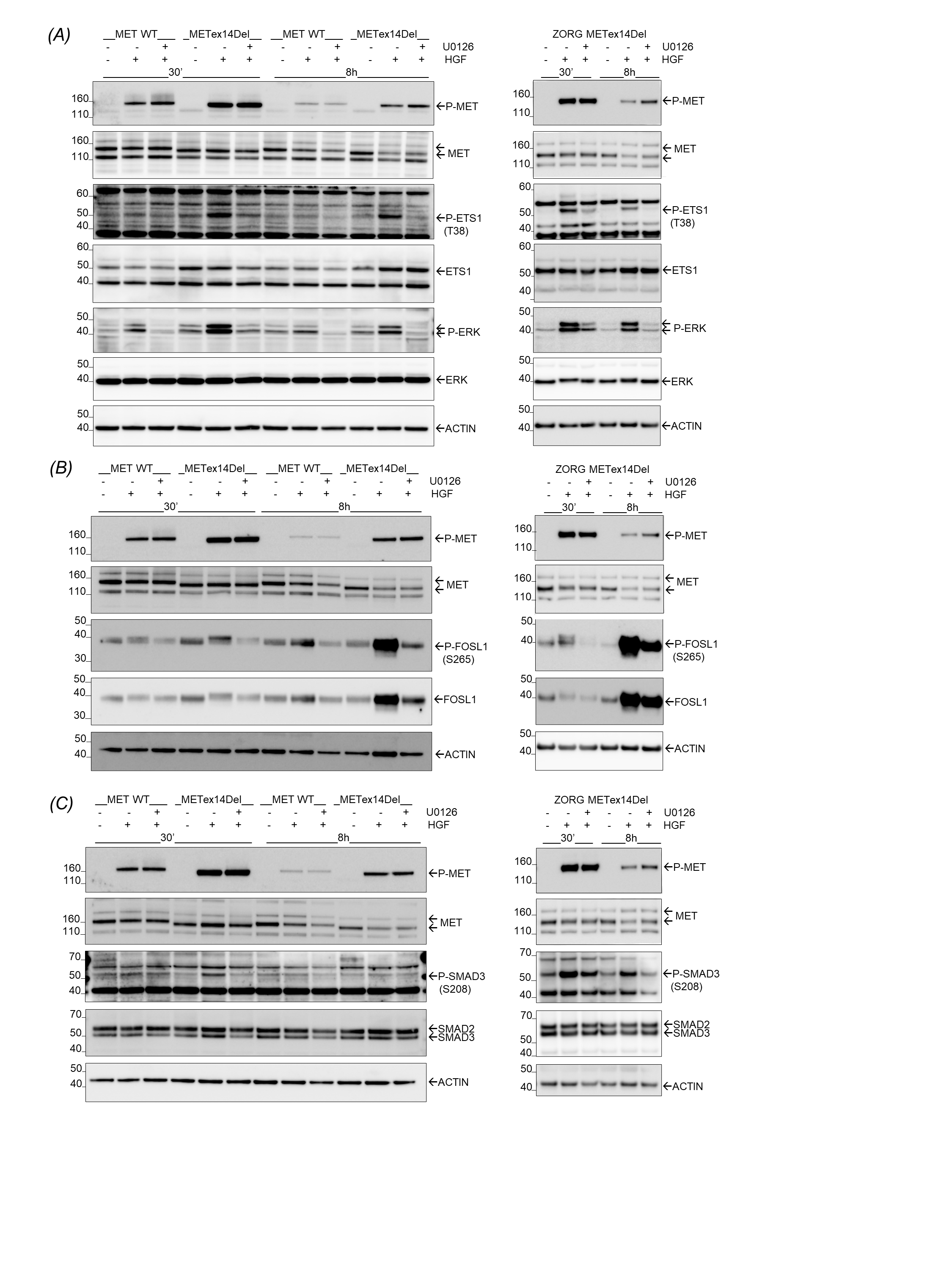

Supplement: Supplementary file 4 — Supplementary Fig. S4 [file 41419_2025_8086_MOESM4_ESM.tif]

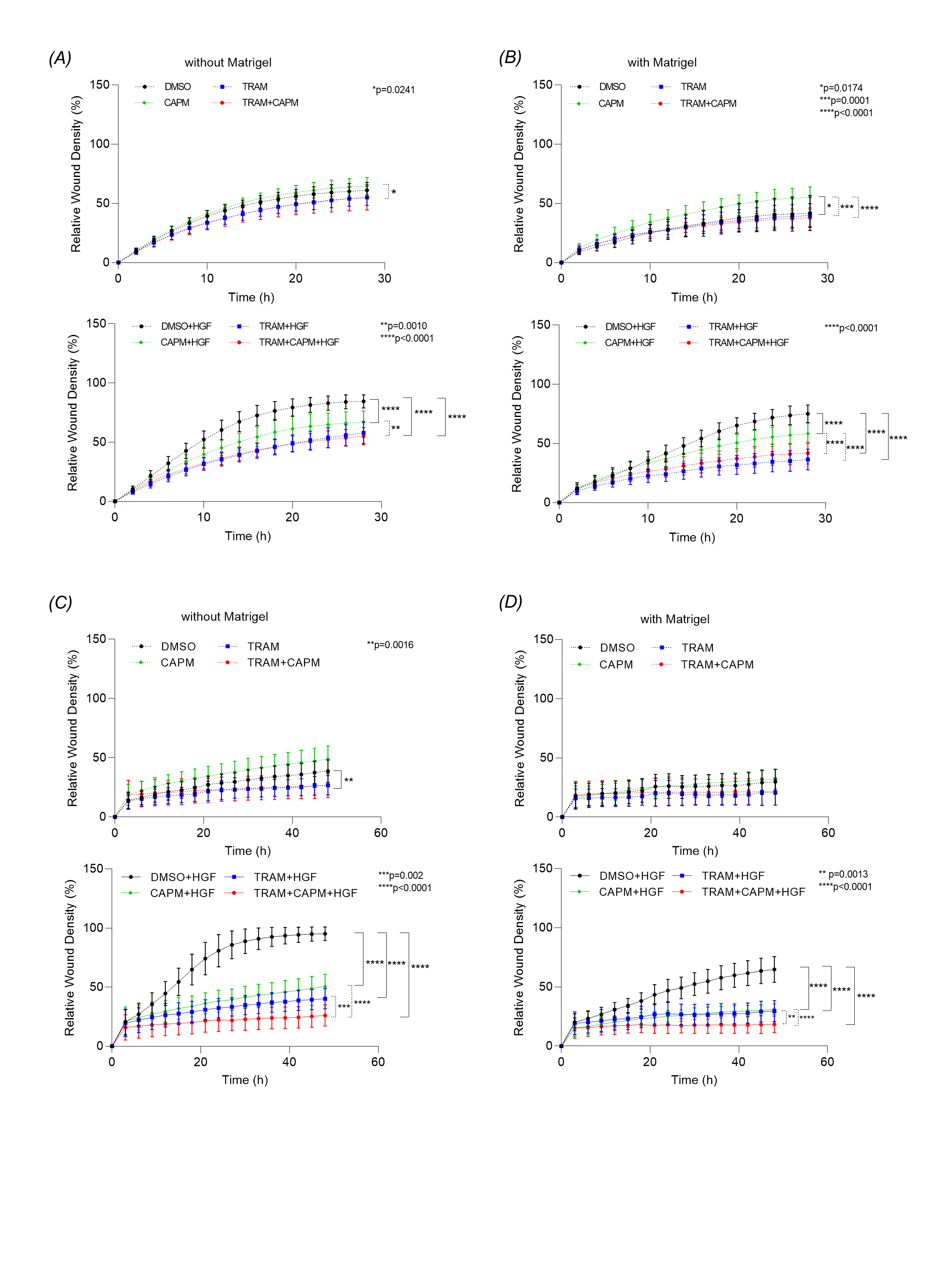

Supplement: Supplementary file 5 — Supplementary Fig. S5 [file 41419_2025_8086_MOESM5_ESM.tif]

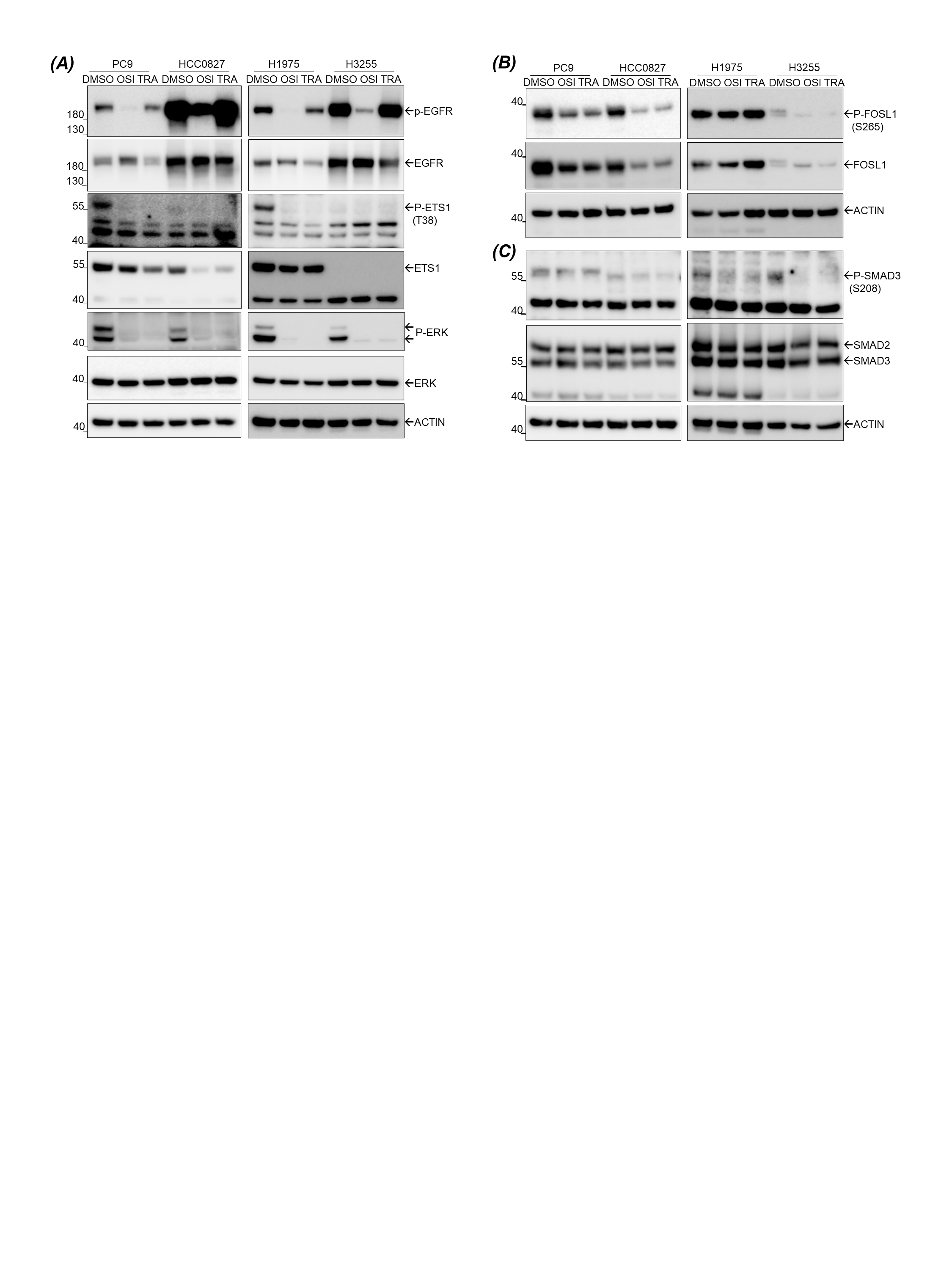

Supplement: Supplementary file 6 — Supplementary Fig. S6 [file 41419_2025_8086_MOESM6_ESM.tif]

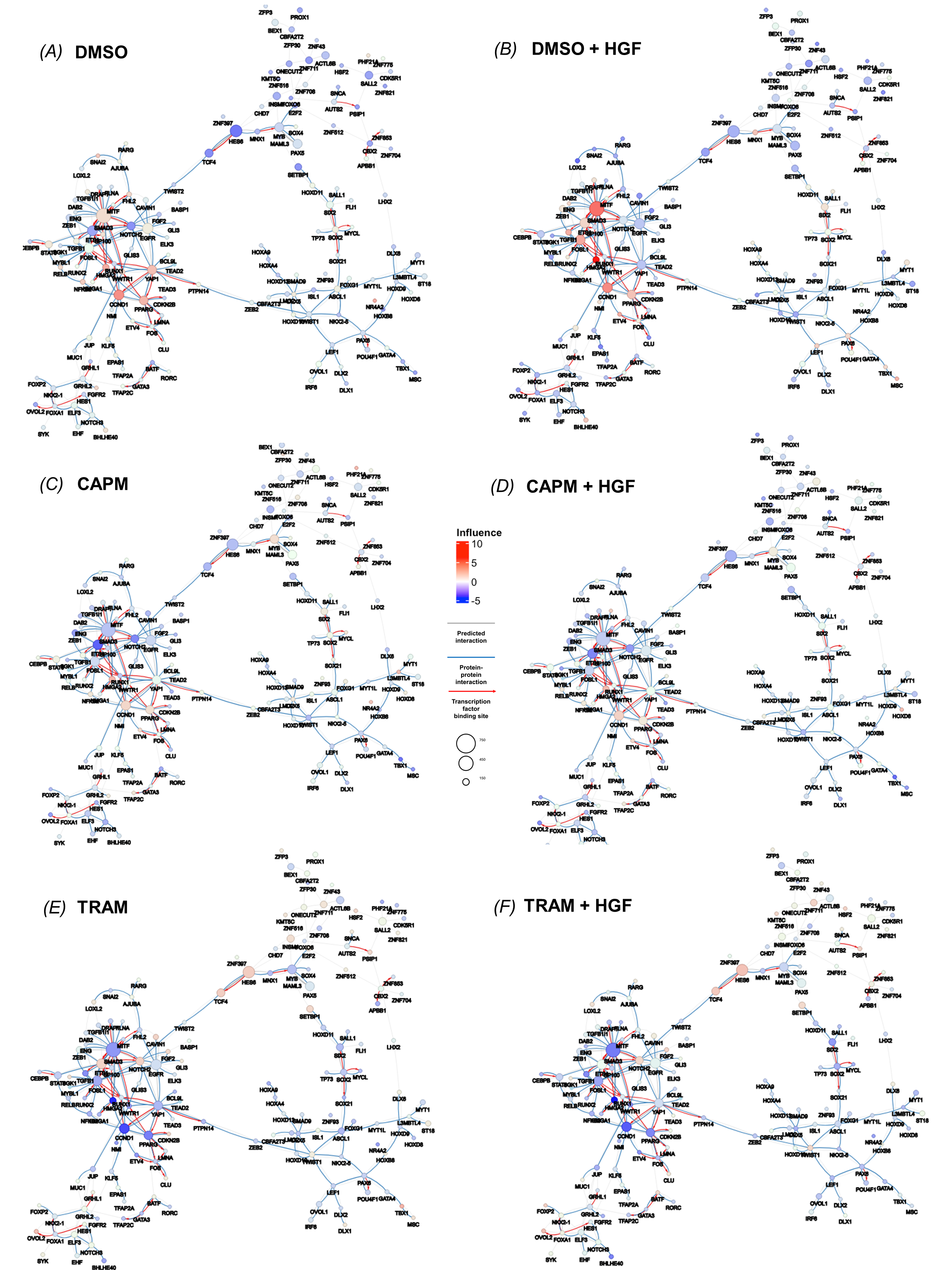

Supplement: Supplementary file 7 — Supplementary Fig. S7 [file 41419_2025_8086_MOESM7_ESM.tif]
