# Supplementary material for: Comprehensive map of the regulatory network triggered by MET exon 14 skipping reveals important involvement of the RAS-ERK signaling pathway
Source: Cell Death Dis. 2025 Nov 3;16(1):783. doi: 10.1038/s41419-025-08086-x (PMC12583649; doi:10.1038/s41419-025-08086-x)
Supplement: Supplementary file 14 — Legends of supplementary figures and tables [file 41419_2025_8086_MOESM14_ESM.docx]

**LEGENDS OF SUPPLEMENTARY FIGURES and TABLES**

**Supplementary Fig. S1** Co-regulatory network showing the influence of TFs on 16HBE MET WT in the absence (**A**) or presence of HGF (**B**) and on 16HBE METex14del in the absence (**C**) or presence of HGF (**D**). Circles represent DIRs and the radius of the circle is proportional to the number of target genes regulated by the DIR. Co-regulatory interactions between DIRs are indicated: protein-protein interactions with published evidence (blue lines), transcriptional regulation interactions with published evidence (red arrows), and interactions defined by the h-LICORN algorithm only (gray lines).

**Supplementary Fig. S2** The mRNA-level expression of additional target genes up- or down-regulated on 16HBE METex14Del in response to HGF was determined by RT-qPCR (triplicates of *n*=4 independent experiments). Significance was determined by unpaired one-tailed *t*-test with Welch’s correction and data are expressed as mean ± S.D. ****p<0.0001

**Supplementary Fig. S3** Time course effect of HGF on protein expression and the corresponding phosphorylation product of (**A**) ETS1, (**B**) FOSL1 and (**C**) SMAD3 in H596 cells according to Western blot (representative results). Time course effect of HGF on mRNA expression of ETS1, FOSL1 and SMAD3 in (**D**) 16HBE MET WT, (**E**) 16HBE METex14Del, (**F**) ZORG and (**G**) H596 cells by RT-qPCR (triplicates of *n*=3 independent experiments). Significance was determined by unpaired one-way ANOVA and data are expressed as mean ± S.D. ****p<0.0001

**Supplementary Fig. S4** Effect of U0126 on the expression of (**A**) P-ETS1/ ETS1, (**B**) P-FOSL1/FOSL1 and (**C**) P-SMAD3/SMAD3 analyzed in 16HBE MET WT, METex14Del cells and ZORG cells stimulated or not with HGF (representative results).

**Supplementary Fig. S5** Effects of trametinib and capmatinib alone and in combination, on cell wound healing without (**A**, **C**) and with (**B**, **D**) Matrigel in lung cancer cell lines: ZORG (**A**, **B**) and H596 (**C**, **D**). In all graphs, only statistically significant differences between the negative control without HGF (DMSO) and the different treatments (individual or combined inhibitors) in the presence or absence of HGF are indicated. Wound healing data in the panels are expressed as mean ± SEM and significance was determined by two-way ANOVA test (6 replicates of *n*=3 independent experiments).

**Supplementary Fig. S6** Effect of MEK inhibitor on ETS1, FOSL1 and SMAD3 phosphorylation. The effect of trametinib (TRA, a MEK inhibitor) on the expression of (**A**) P-ETS1/ETS1, (**B**) P-FOSL1/FOSL1 and (**C**) P-SMAD3/SMAD3 in EGFR-driven lung cancers (PC9, HCC0827, H1975 and H3255 cell lines) was determined by Western blotting (representative results).

**Supplementary Fig. S7** Co-regulatory network showing the influence of TFs on 16HBE METex14Del cells without treatment in the absence (**A**) or presence (**B**) of HGF; or with treatment by capmatinib (CAPM) in the absence (**C**) or presence (**D**) of HGF; or with treatment by trametinib (TRAM) in the absence (**E**) or presence (**F**) of HGF. Circles represent DIRs and the radius of the circle is proportional to the number of target genes regulated by the DIR. Co-regulatory interactions between DIRs are indicated: protein-protein interactions with published evidence (blue lines), transcriptional regulation interactions with published evidence (red lines), and interactions defined only by the h-LICORN algorithm (gray lines).

**Supplementary Table 1.** Influence of key differentially acting regulators (METex14Del+HGF vs. resting conditions)

**Supplementary Table 2.** Genes targeted by ETS1, FOSL1 and SMAD3

**Supplementary Table 3.** Gene Ontology Enrichment for the differentially expressed target genes

**Supplementary Table 4.** Mean Influence (MI) of regulators in DMSO, Capmatinib or Trametinib treatment under HGF stimulation conditions

**Supplementary Table 5.** List of products

**Supplementary Table 6.** Primers sequences used for qPCR
