# Supplementary material for: Comprehensive map of the regulatory network triggered by MET exon 14 skipping reveals important involvement of the RAS-ERK signaling pathway
Source: Cell Death Dis. 2025 Nov 3;16(1):783. doi: 10.1038/s41419-025-08086-x (PMC12583649; doi:10.1038/s41419-025-08086-x)

Fig.3C

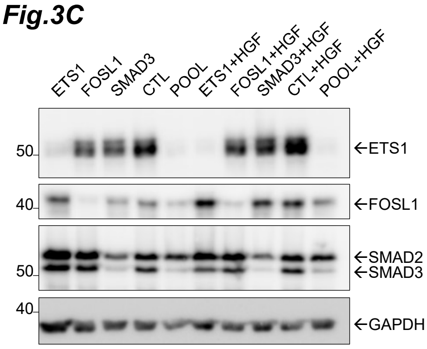
ETS1
FOSL1
SMAD3
CTL
POOL
ETS1+HGF
FOSL1+HGF
SMAD3+HGF
CTL+HGF
POOL+HGF
50
←ETS1
40
←FOSL1
←SMAD2
50
←SMAD3
40
←GAPDH

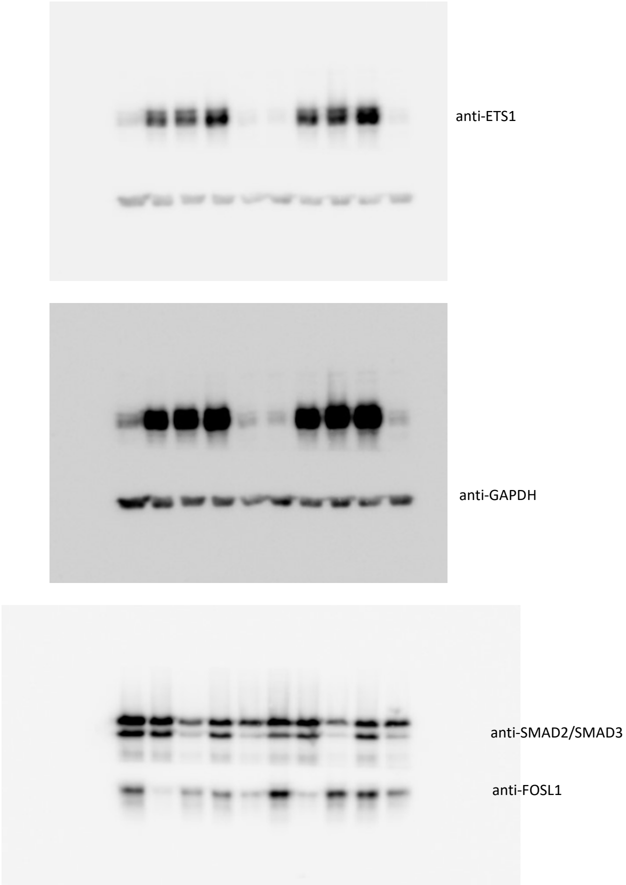
anti-ETS1
anti-GAPDH
anti-SMAD2/SMAD3
anti-FOSL1

Fig. 5C

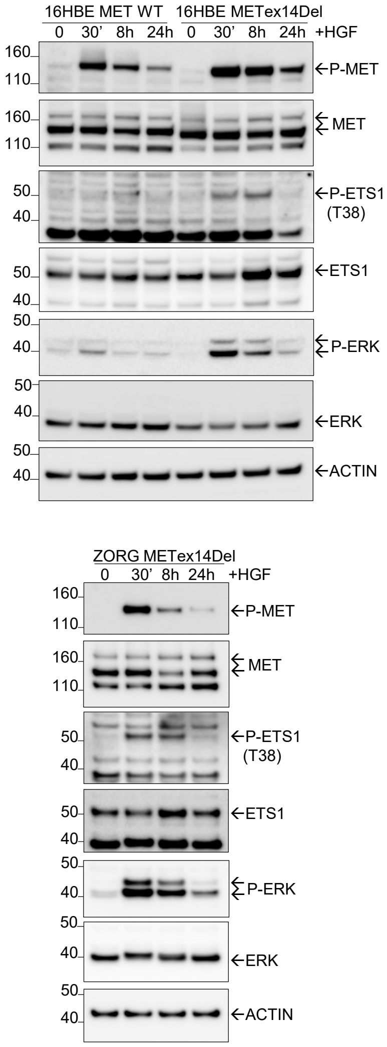
16HBE MET WT
16HBE METex14Del
0 30' 8h 24h 0 30' 8h 24h +HGF
160
110
P-MET
MET
50
40
P-ETS1
(T38)
ETS1
P-ERK
ERK
ACTIN
ZORG METex14Del
0 30' 8h 24h +HGF

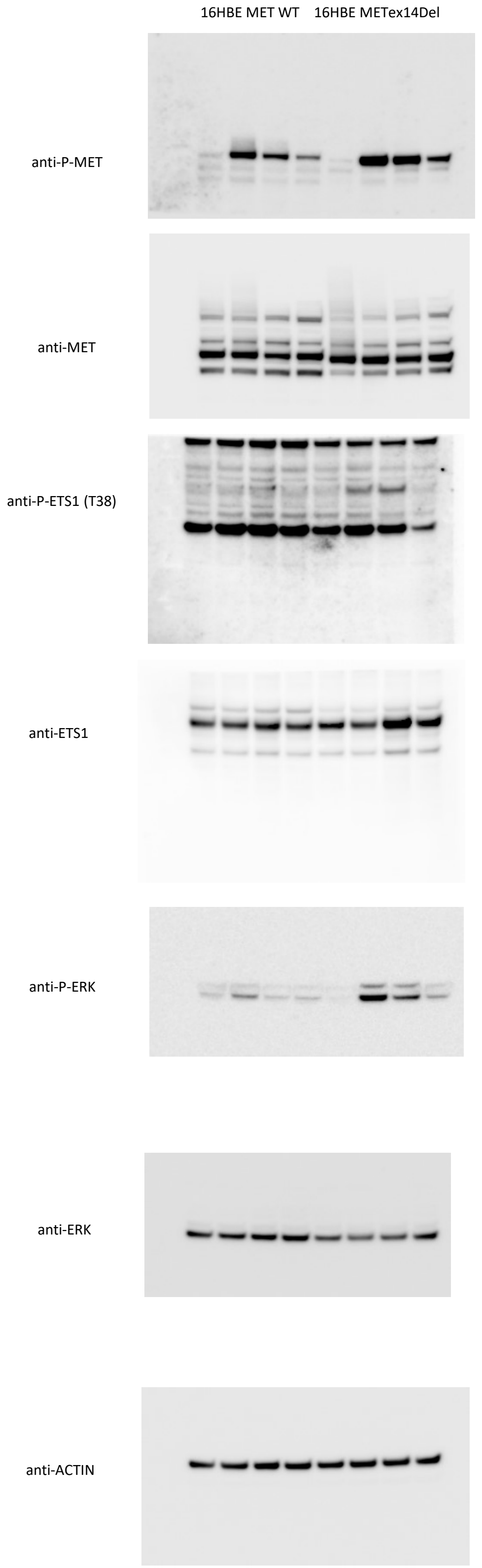
16HBE MET WT
16HBE METex14Del
anti-P-MET
anti-MET
anti-P-ETS1 (T38)
anti-ETS1
anti-P-ERK
anti-ERK
anti-ACTIN

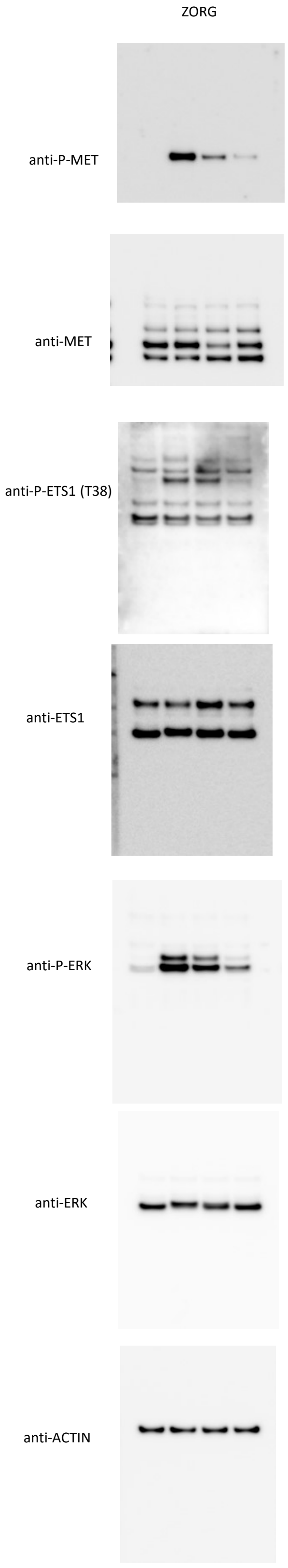
ZORG
anti-P-MET
anti-MET
anti-P-ETS1 (T38)
anti-ETS1
anti-P-ERK
anti-ERK
anti-ACTIN

Fig. 5D

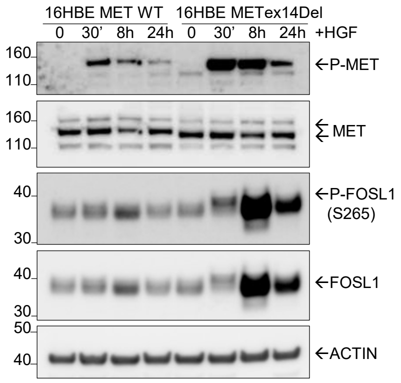
16HBE MET WT
16HBE METex14Del
0 30' 8h 24h 0 30' 8h 24h +HGF
160
110
P-MET
160
110
MET
40
30
P-FOSL1
(S265)
40
30
FOSL1
50
40
ACTIN

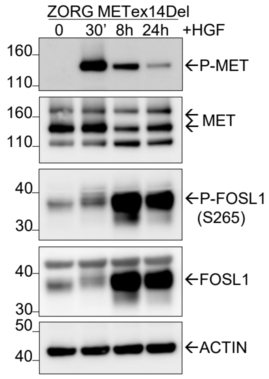
ZORG METex14Del
0 30' 8h 24h +HGF
160
110
P-MET
160
110
MET
40
30
P-FOSL1
(S265)
40
30
FOSL1
50
40
ACTIN

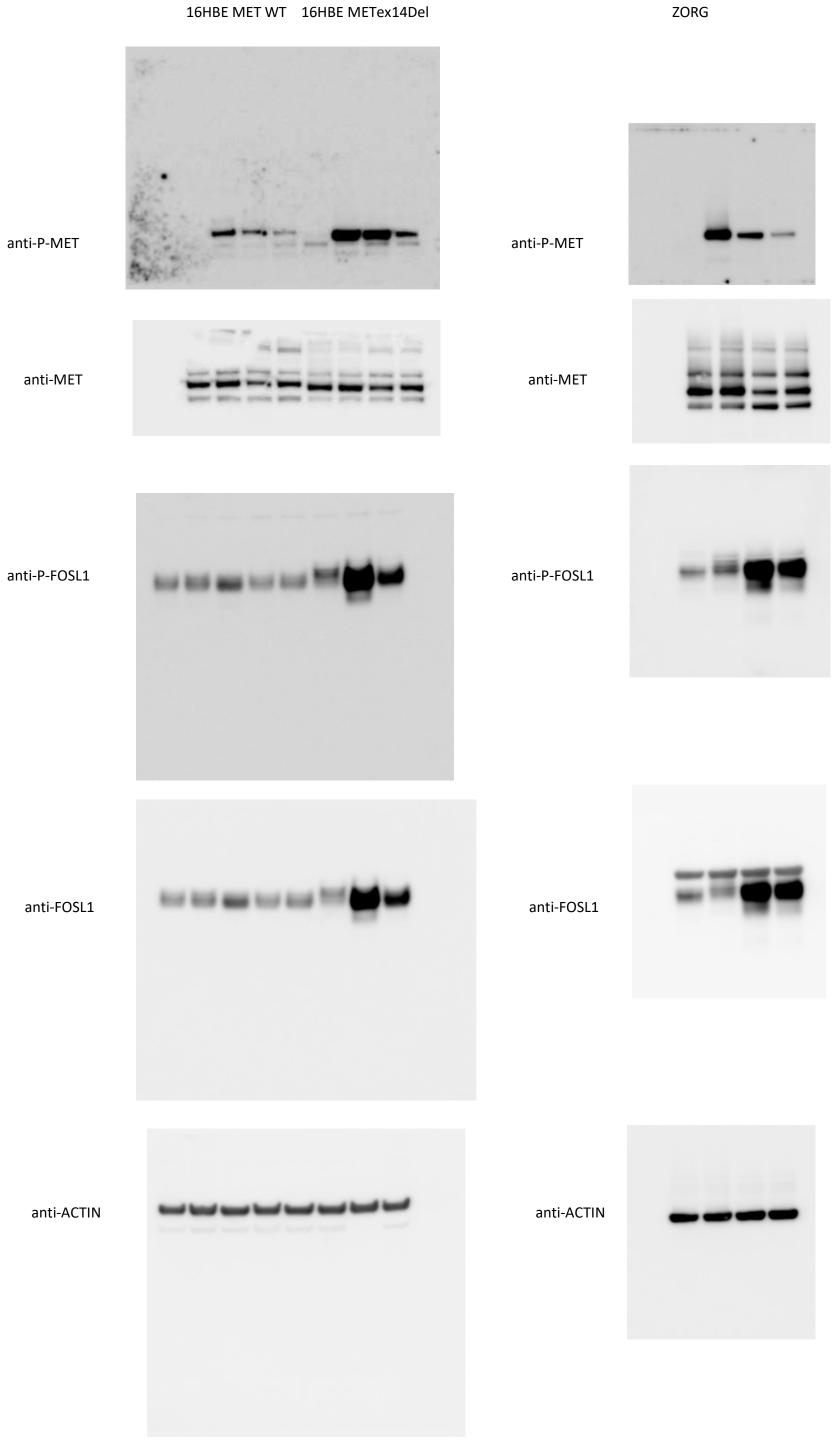
16HBE MET WT
16HBE METex14Del
ZORG
anti-P-MET
anti-P-MET
anti-MET
anti-MET
anti-P-FOSL1
anti-P-FOSL1
anti-FOSL1
anti-FOSL1
anti-ACTIN
anti-ACTIN

Fig. 5E

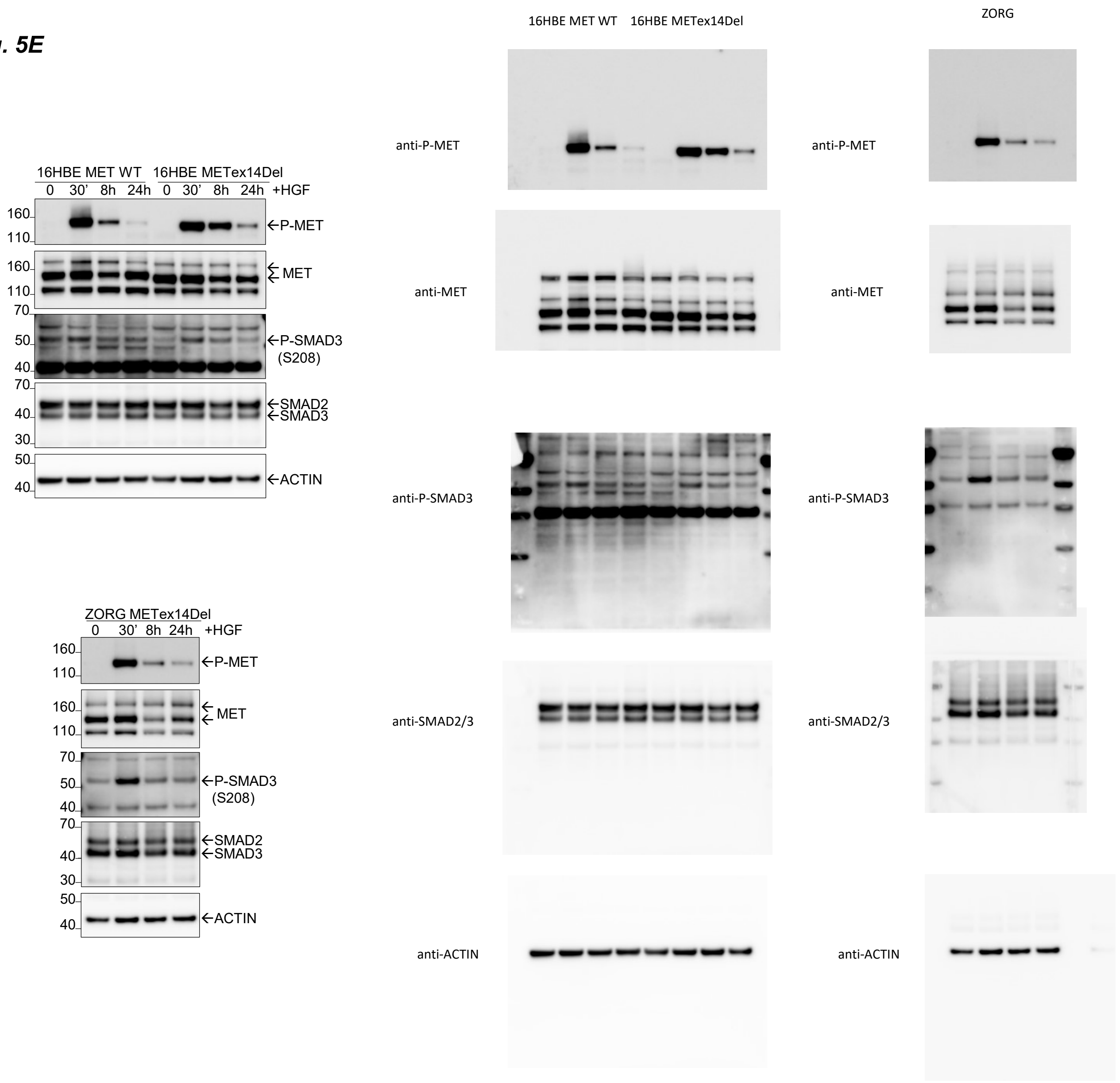
16HBE MET WT
16HBE METex14Del
0
30'
8h
24h
+HGF
160
110
P-MET
MET
70
50
40
P-SMAD3
(S208)
30
SMAD2
SMAD3
ACTIN
ZORG METex14Del
ZORG
anti-P-MET
anti-MET
anti-P-SMAD3
anti-SMAD2/3
anti-ACTIN

Fig. 6A

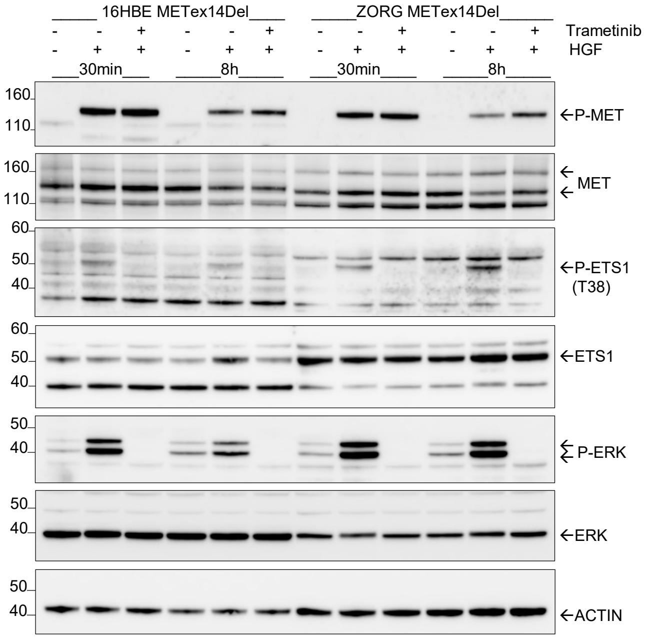
16HBE METex14Del
ZORG METex14Del
Trametinib
HGF
30min
8h
30min
8h
160
110
←P-MET
160
110
MET
60
50
40
←P-ETS1 (T38)
60
50
40
←ETS1
50
40
P-ERK
50
40
←ERK
50
40
←ACTIN

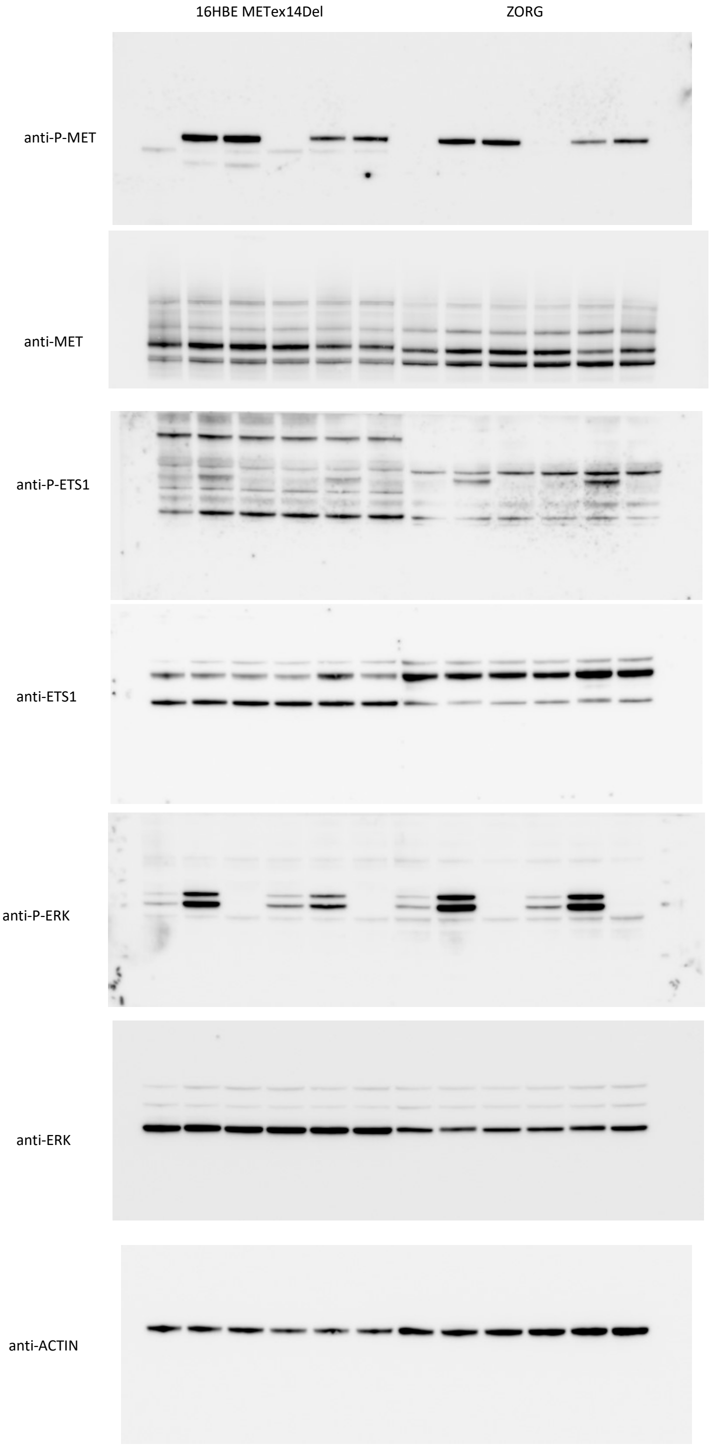
16HBE METex14Del
ZORG
anti-P-MET
anti-MET
anti-P-ETS1
anti-ETS1
anti-P-ERK
anti-ERK
anti-ACTIN

Fig. 6B

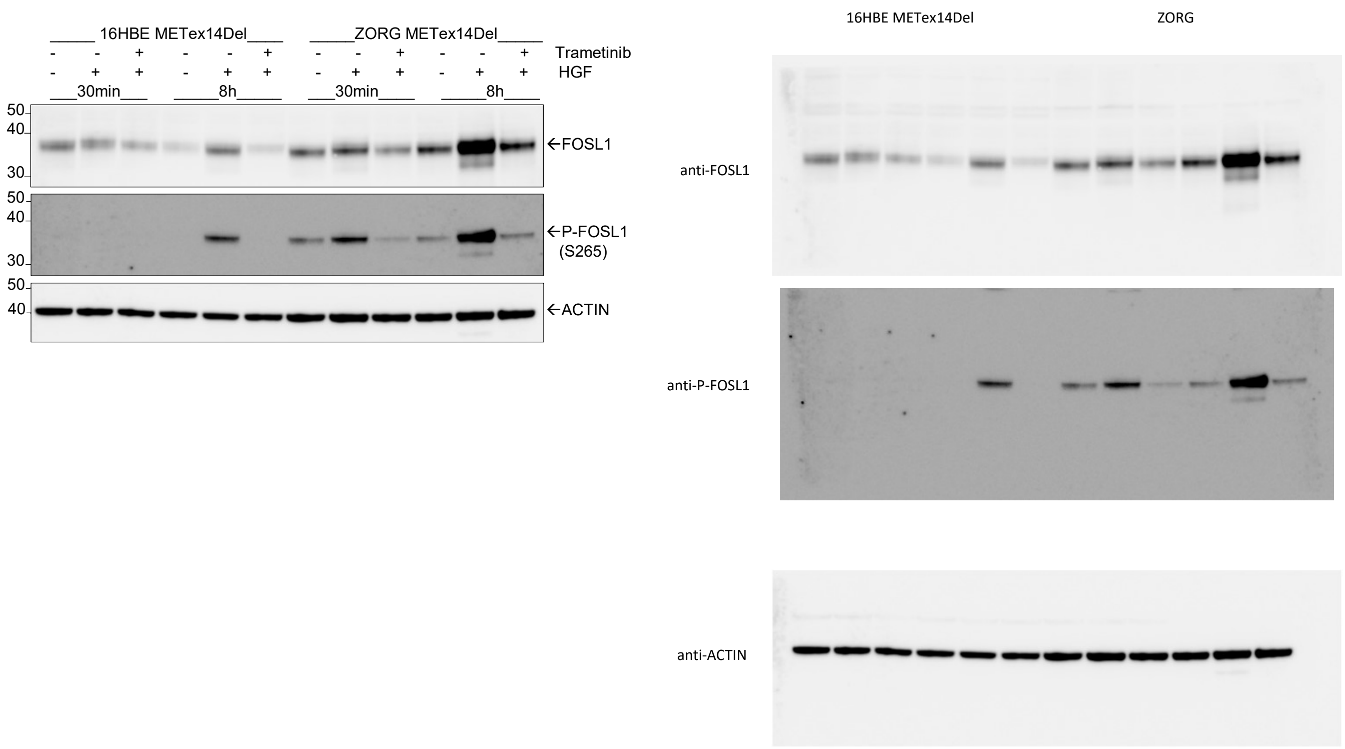
16HBE METex14Del
ZORG METex14Del
Trametinib
HGF
30min
8h
30min
8h
50
40
30
50
40
30
50
40
←FOSL1
←P-FOSL1
(S265)
←ACTIN
16HBE METex14Del
ZORG
anti-FOSL1
anti-P-FOSL1
anti-ACTIN

Fig. 6C

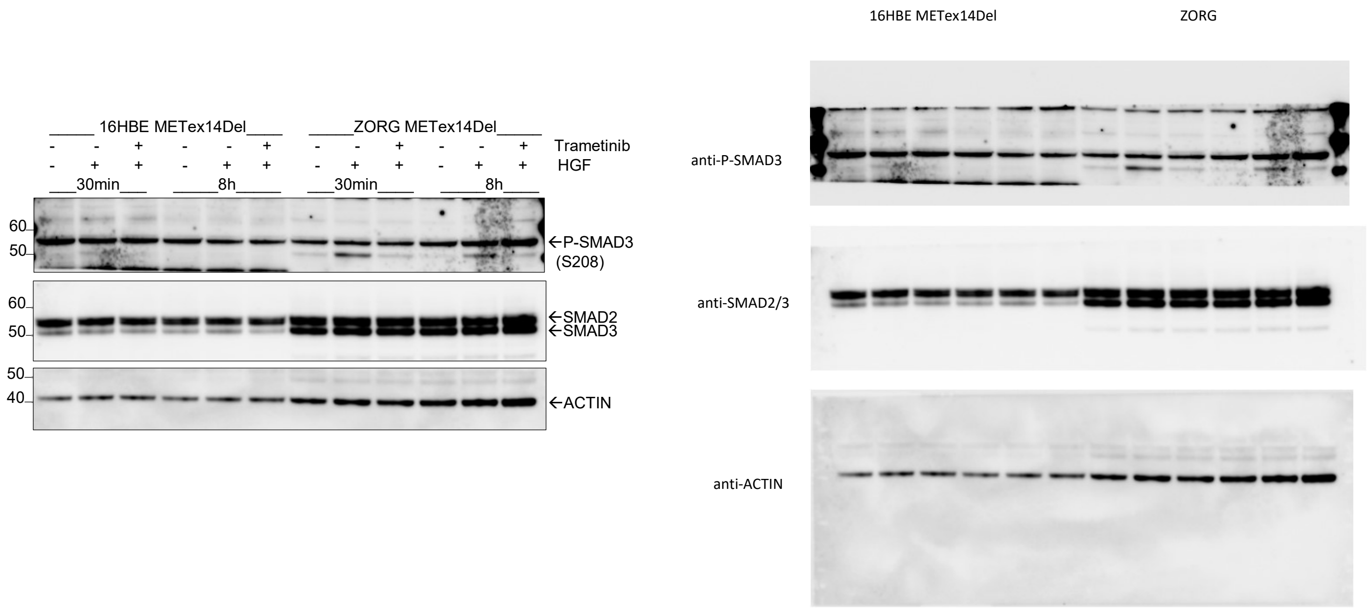
16HBE METex14Del
ZORG METex14Del
Trametinib
HGF
30min
8h
30min
8h
60
50
60
50
50
40
←P-SMAD3
(S208)
←SMAD2
←SMAD3
←ACTIN
16HBE METex14Del
ZORG
anti-P-SMAD3
anti-SMAD2/3
anti-ACTIN

Supplementary Fig 3

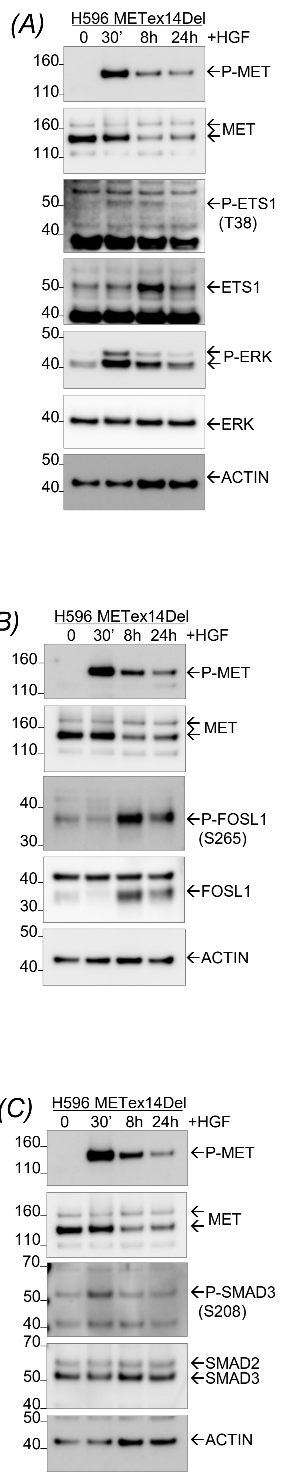

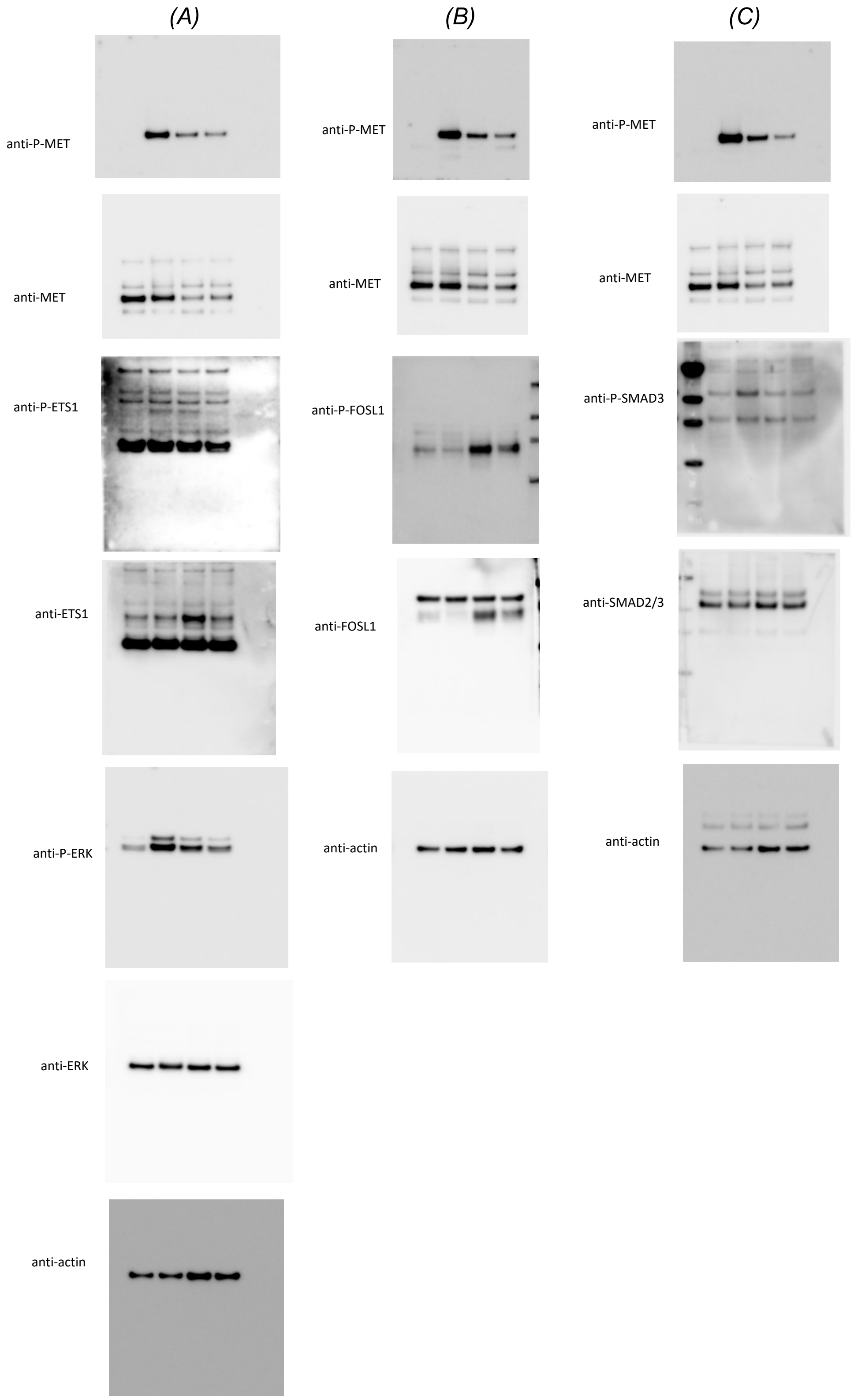

Supplementary Fig 4A 16HBE

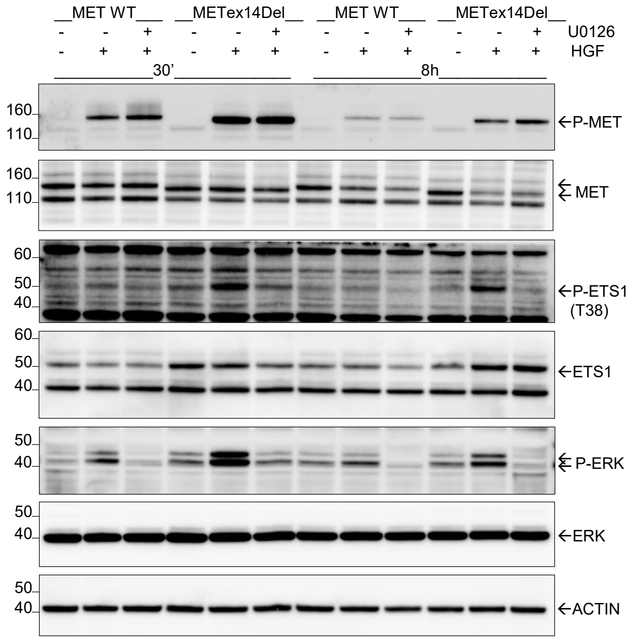

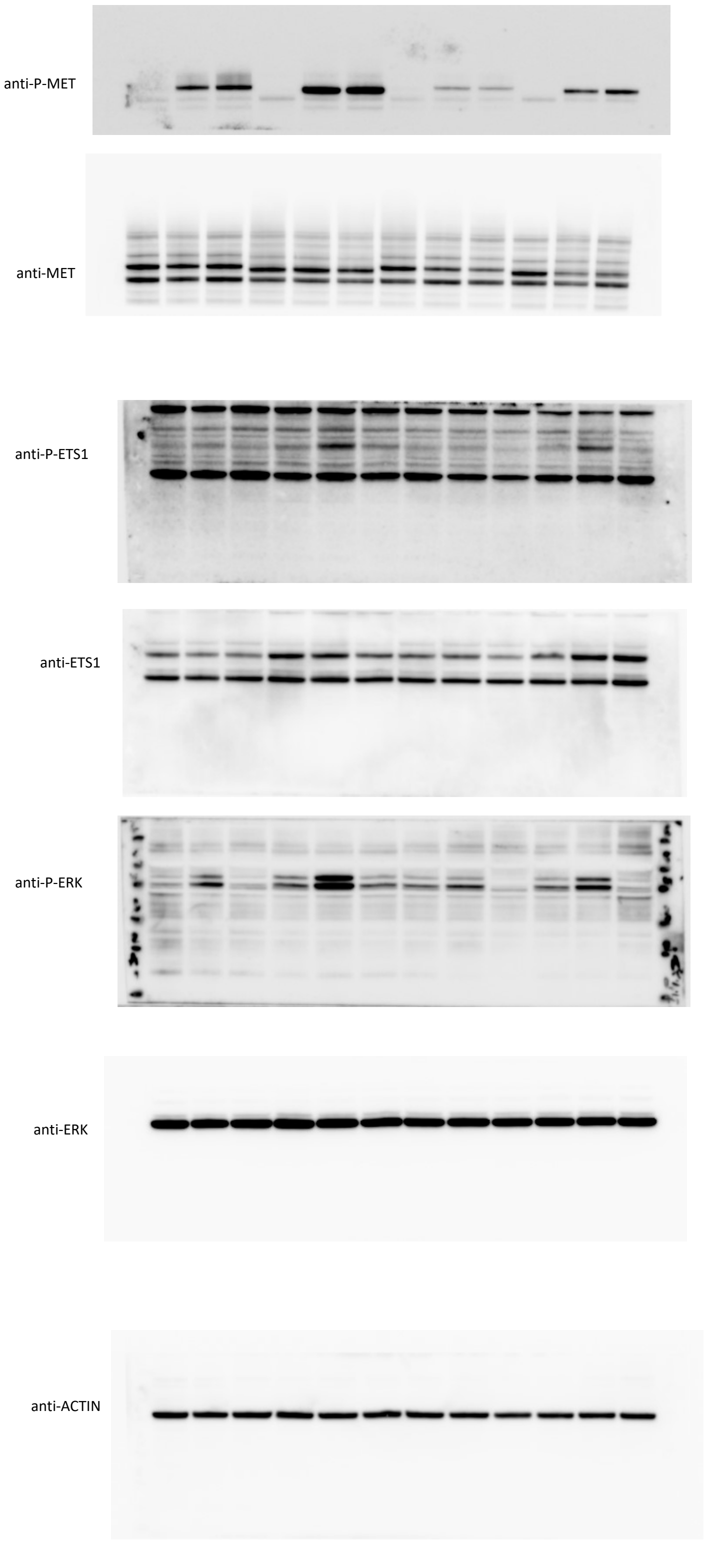

Supplementary Fig 4B 16HBE

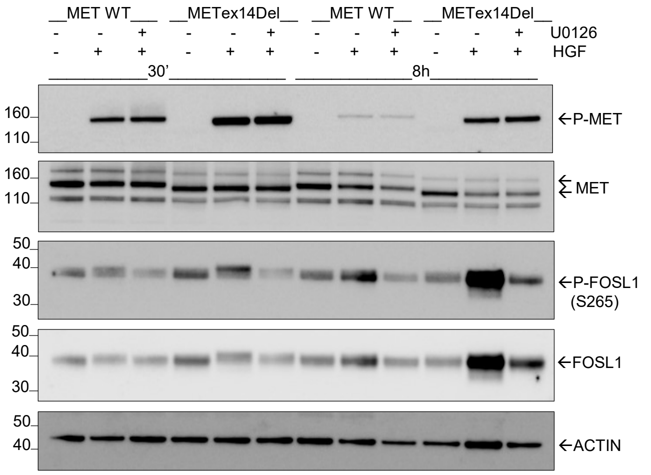

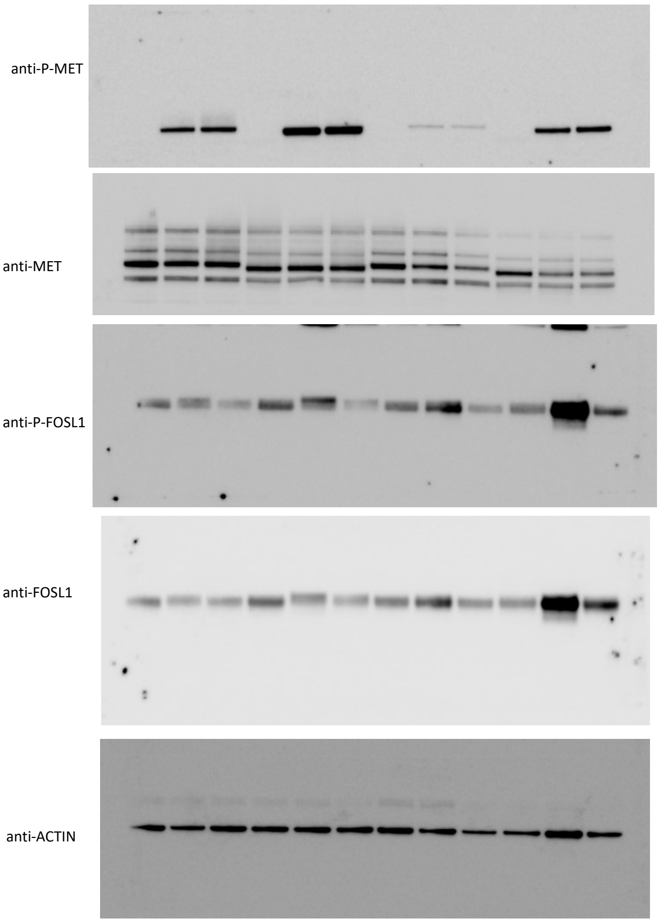

Supplementary Fig 4C 16HBE

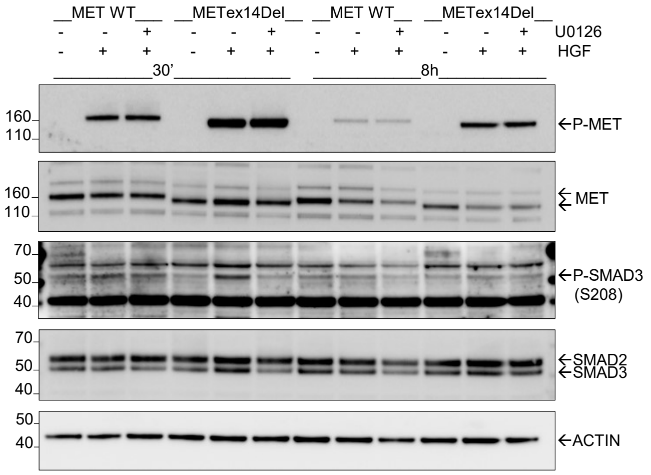

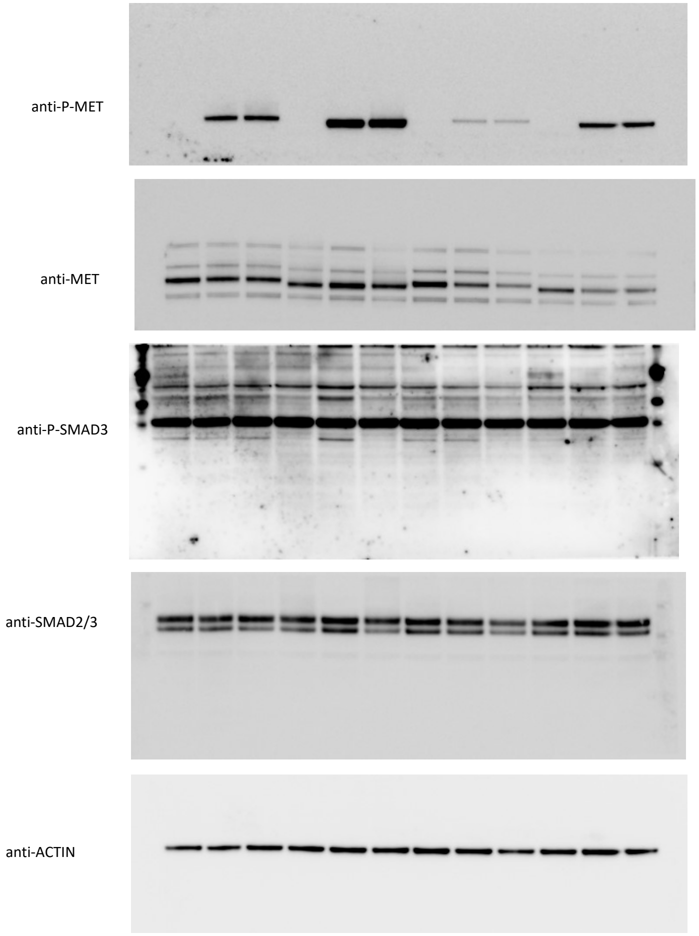

Supplementary Fig 4 ZORG

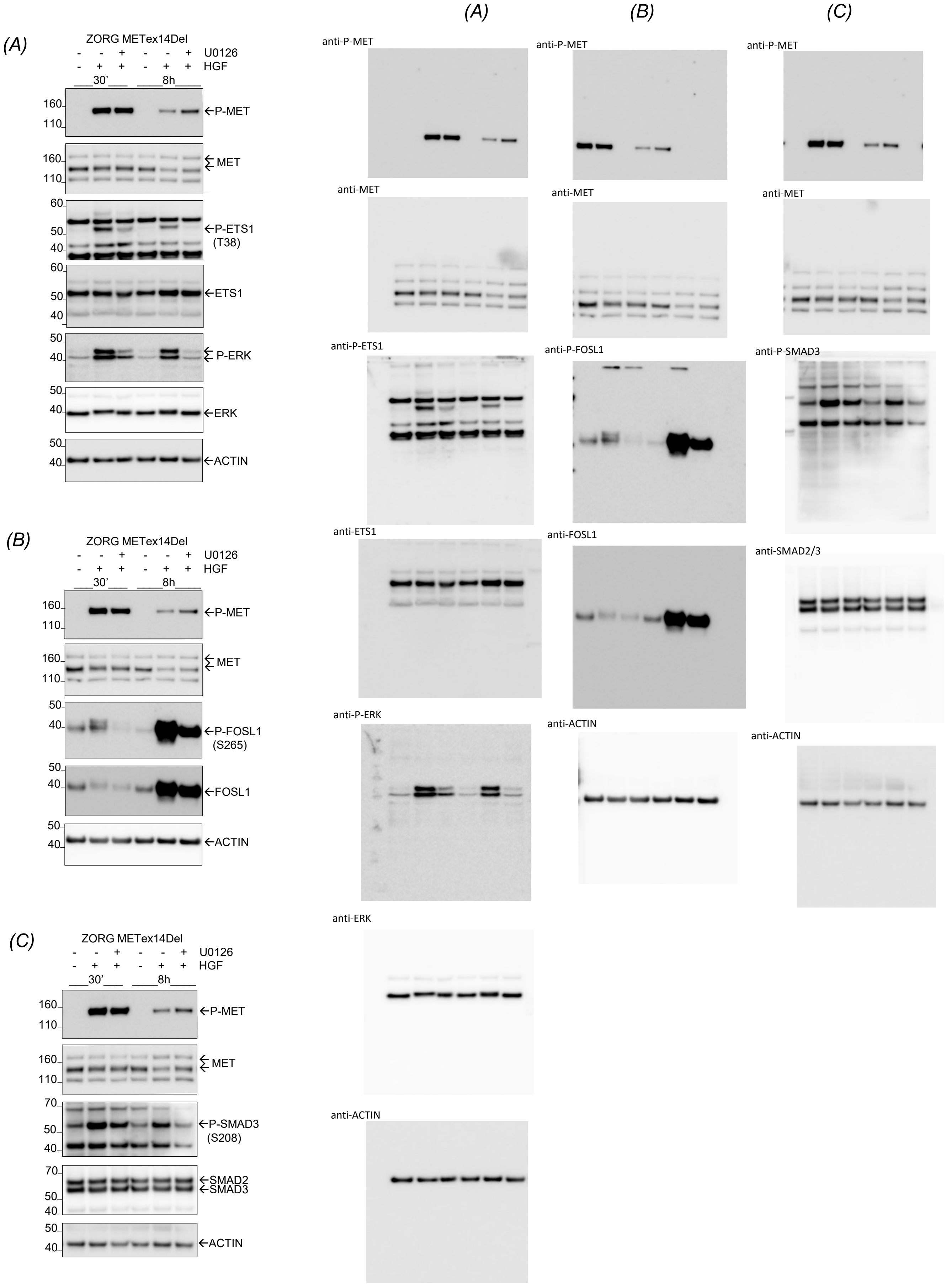

Supplementary Fig 6A PC9 HCC827 H1975 H3255

(A)

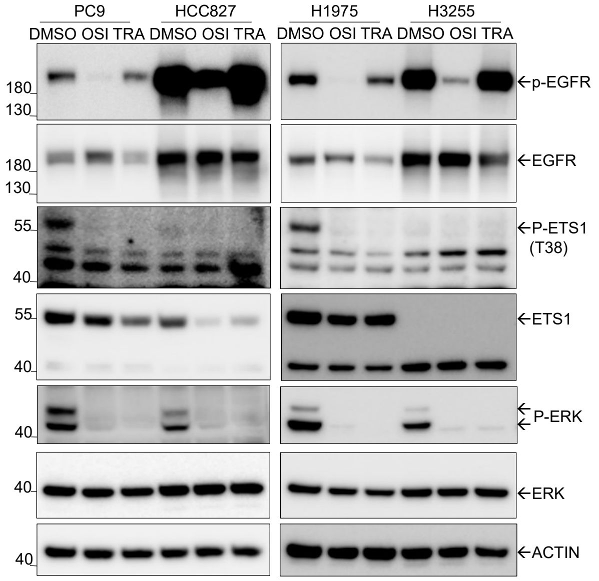

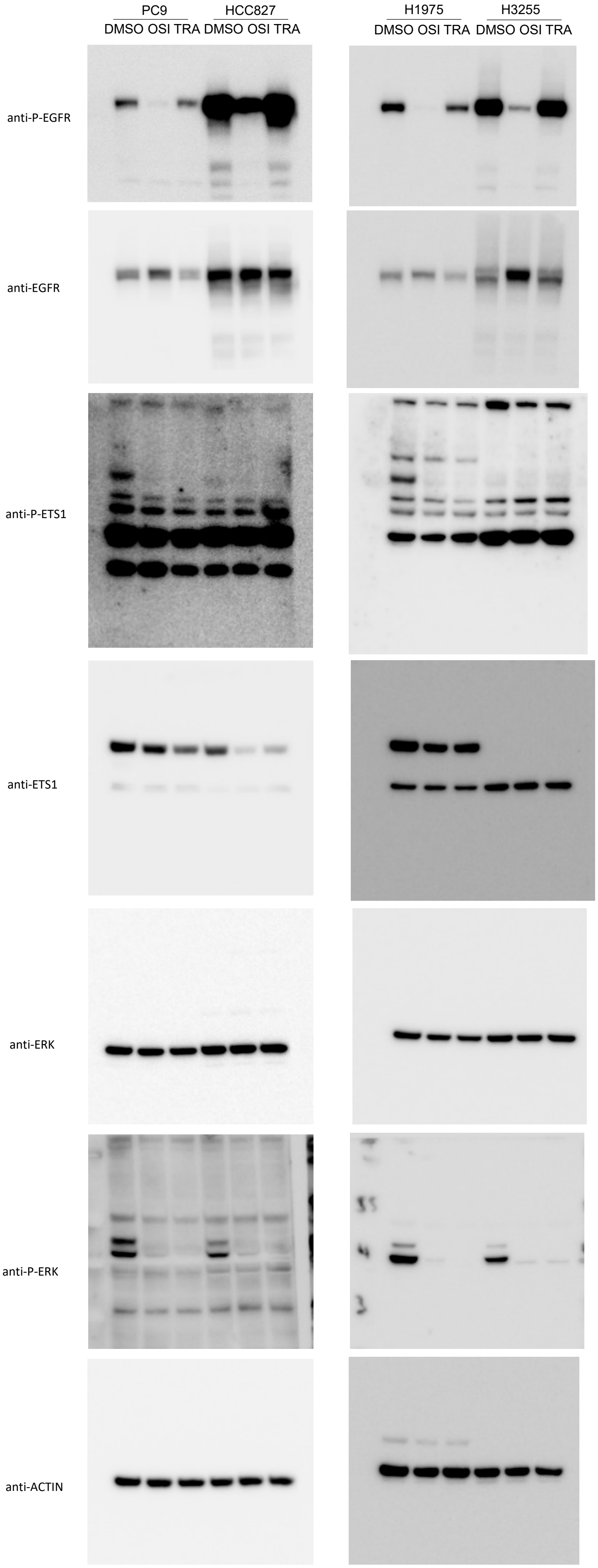

Supplementary Fig 6(B-C) PC9 HCC827 H1975 H3255

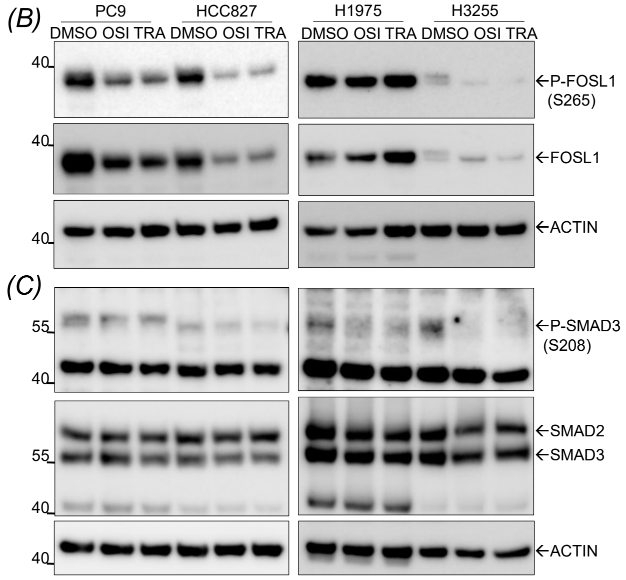

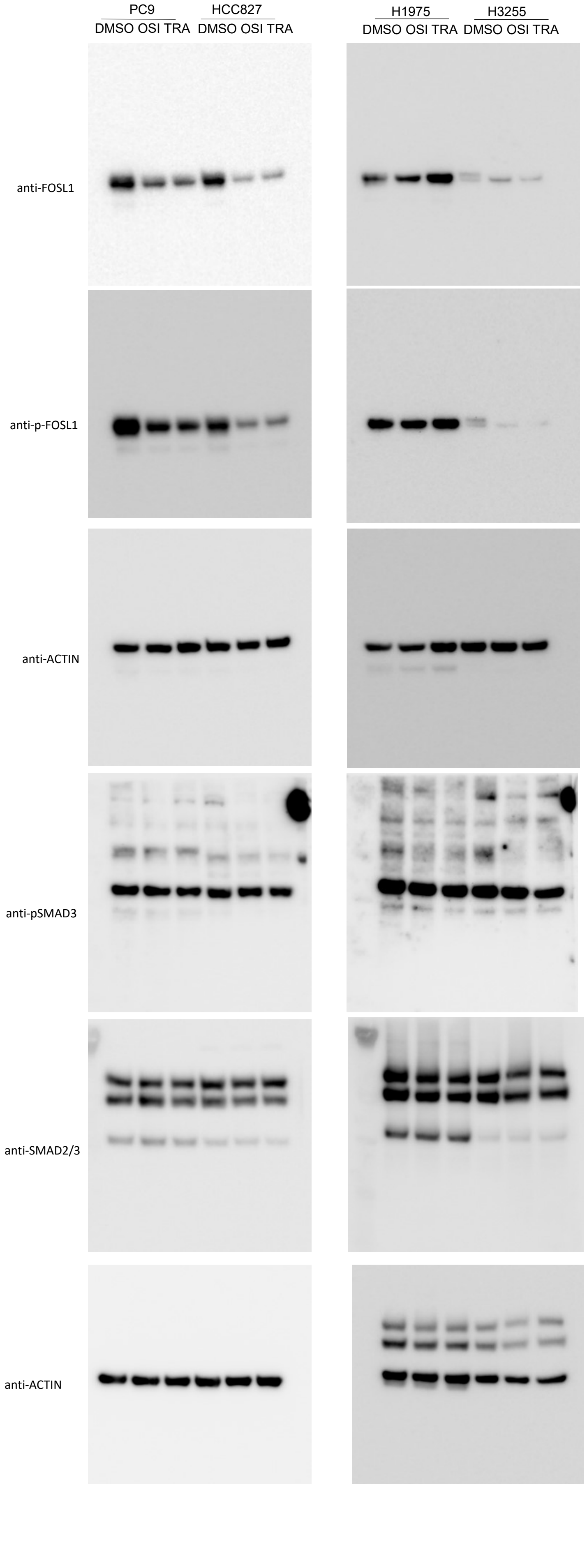

Supplement: Supplementary file 15 — revised original western blots [file 41419_2025_8086_MOESM15_ESM.pdf]
